# Rational design of pigment–polymer antenna complexes

**DOI:** 10.1039/d6sc01202g

**Published:** 2026-04-24

**Authors:** Edwin C. Johnson, Demetris Bates, Tingxiang Yang, Kasimir Gregory, Jodie West, Deborah B. Hammond, James P. Pidgeon, Jenny Clark, Nicholas H. Williams, C. Neil Hunter, Steven P. Armes, Graham J. Leggett

**Affiliations:** a School of Mathematical and Physical Sciences, University of Sheffield Dainton Building, Brook Hill Sheffield S3 7HF UK Graham.Leggett@sheffield.ac.uk; b School of Biosciences, University of Sheffield Firth Court Sheffield S10 2TN UK; c School of Science and Technology, University of New England Armidale NSW 2351 Australia

## Abstract

We report the synthesis of biomimetic programmable pigment–polymer antenna complexes (PPACs) by using reductive amination to bind amine-functional dyes to surface-grafted aldehyde-functional PAGEO5MA chains grown from planar substrates by atom-transfer radical polymerization. The fraction of dye-conjugated repeat units can approach unity under optimized conditions. Dye binding kinetics are strongly influenced by steric factors and can be controlled by varying the polymer grafting density, the dye size and the nucleophilicity of its amine group. Absorption and fluorescence spectra of PPACs produced by conjugating Nile Red ethylamine (NRet) to PAGEO5MA brushes are sensitive to the dielectric environment within the layer. At low dye concentrations, the mean fluorescence lifetime *τ*_mean_ of the chromophore is 1.3 ± 0.1 ns, similar to that obtained for a dilute methanolic solution of NRet (1.17 ± 0.01 ns). *τ*_mean_ decreases with increasing dye conjugation, due to increased dye–dye interactions. However, *τ*_mean_ is higher for NRet conjugated to PAGEO5MA than for NR in a spin-cast film of the dye in poly(methyl methacrylate) at the same concentration, indicating that conjugation to the polymer scaffold minimizes dye aggregation. PPACs offer a potentially versatile route to the production of programmable photonic materials, with efficient conjugation chemistry enabling precise control over dye–dye interactions.

## Abstract 2

Programmable pigment–polymer complexes are produced *via* conjugation of amine-functionalized dyes to aldehyde-functionalized brushes. Dye conjugation to a brush enables control of dye–dye interactions, greatly extending the excited state lifetime.

## Graphical abstract

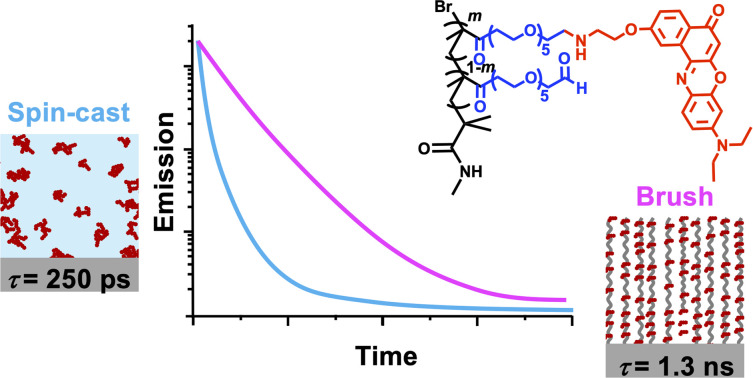

## Introduction

Absorption of a photon by a molecule leads to the formation of an electron–hole pair, or exciton. There is growing interest in the use of molecular materials to develop sustainable approaches to the design of optoelectronic devices.^1,2^ Molecular photonic materials can be produced from Earth-abundant elements using low energy processes, so in principle they offer the potential for minimal environmental impact.^3^ Organic semiconductors are now widely used in display technologies.^4,5^ However, their application in other technologies, for example solar energy generation, has been impeded by a lack of control over exciton dynamics, which are dominated by incoherent hopping processes.^1,3^ For organic semiconductors, exciton diffusion lengths are typically ∼10 nm (exceptionally a few tens of nm), which places significant constraints on device fabrication. The development of robust design rules for the construction of molecular materials that enable efficient, long-range transport of excitons remains an unsolved grand challenge.^6^

Photosynthetic pigment–protein light-harvesting complexes (LHCs) isolated from plants and bacteria achieve high fluorescence quantum yields.^7–10^ They utilize peptide scaffolds for the precise three-dimensional (3D) organization of pigment molecules (carotenoids and chlorophylls), achieving high concentrations for such pigments (∼0.3 M in plant-based LHCs) with minimal quenching of excited states. Thus, they provide an attractive paradigm for the design of molecular photonic materials. Recently, we explored a new approach to the design of programmable photonic materials involving the use of surface-grafted poly(amino acid methacrylate) chains formed by surface-initiated atom-transfer radical polymerization (SI-ATRP) to organize chlorophyll within a 3D structure.^11,12^ Chlorophyll could be efficiently conjugated to these synthetic polymer scaffolds to achieve higher pigment concentrations than those found in LHCs. Moreover, the properties of the resulting 3D structures could be manipulated by controlling the brush grafting density. When these pigment–polymer complexes (PPACs) were prepared using gold nanostructure arrays, strong plasmon–exciton coupling was observed, with coupling energies approximately twice as high as those reported previously for photosynthetic LHCs.^13–15^ This is consistent with the relatively high chlorophyll concentrations within such PPACs.^11^

Although this new approach is very promising, chlorophyll has rather limited solubility in many common solvents potentially limiting its compatibility with other materials and processes. Moreover, a wide range of reactive dyes are required to target specific regions of the electromagnetic spectrum and hence provide greater programmability. A versatile yet generic approach to the design of PPACs based on dye-functionalized polymer brushes requires efficient conjugation chemistry to ensure minimization of dye aggregation and hence optimization of the photonic properties of the film.

There is a substantial body of literature on dye conjugation to polymer brushes.^16–21^ However, in most cases dye functionalization occurs at the end-group or the degree of dye incorporation is relatively low, with a dye/repeat unit molar ratio <0.10.^17,22^ In contrast, it is essential to achieve high concentrations of bound dye when designing photonic materials. For polymer brushes, the two principal synthetic challenges are (i) the steric constraints that limit penetration of a swollen brush layer by relatively large dye molecules and (ii) identification of a suitable solvent that ensures a highly swollen brush layer while also enabling a high degree of dye conjugation to be achieved.^22,23^

Recently we described the synthesis of poly(glycerol penta(ethylene oxide)methacrylate) (PGEO5MA) brushes by surface-initiated atom transfer radical polymerization (SI ATRP).^24^ Subsequently this *cis*-diol precursor was converted into the corresponding hydrophilic aldehyde-functional brush, herein denoted as PAGEO5MA,^24,25^*via* selective oxidation of the pendant *cis*-diol groups. Untethered PAGEO5MA homopolymer, various types of PAGEO5MA-based diblock copolymer nanoparticles and PAGEO5MA brushes have each been shown to be capable of chemical conjugation with various reactive amines.^24–30^ The commercial availability of many amine-functional dyes and the relative ease by which amine groups can be introduced to dye molecules make such surface-grafted PAGEO5MA films attractive candidates for the design of PPACs with programmable excitonic properties. To achieve this aim, it is important to understand how the polymer/solvent interactions, dye conjugation chemistry, dye loading and brush grafting density influence the spectroscopic properties of the dye-conjugated brush layer.^22^

**Scheme 1:**
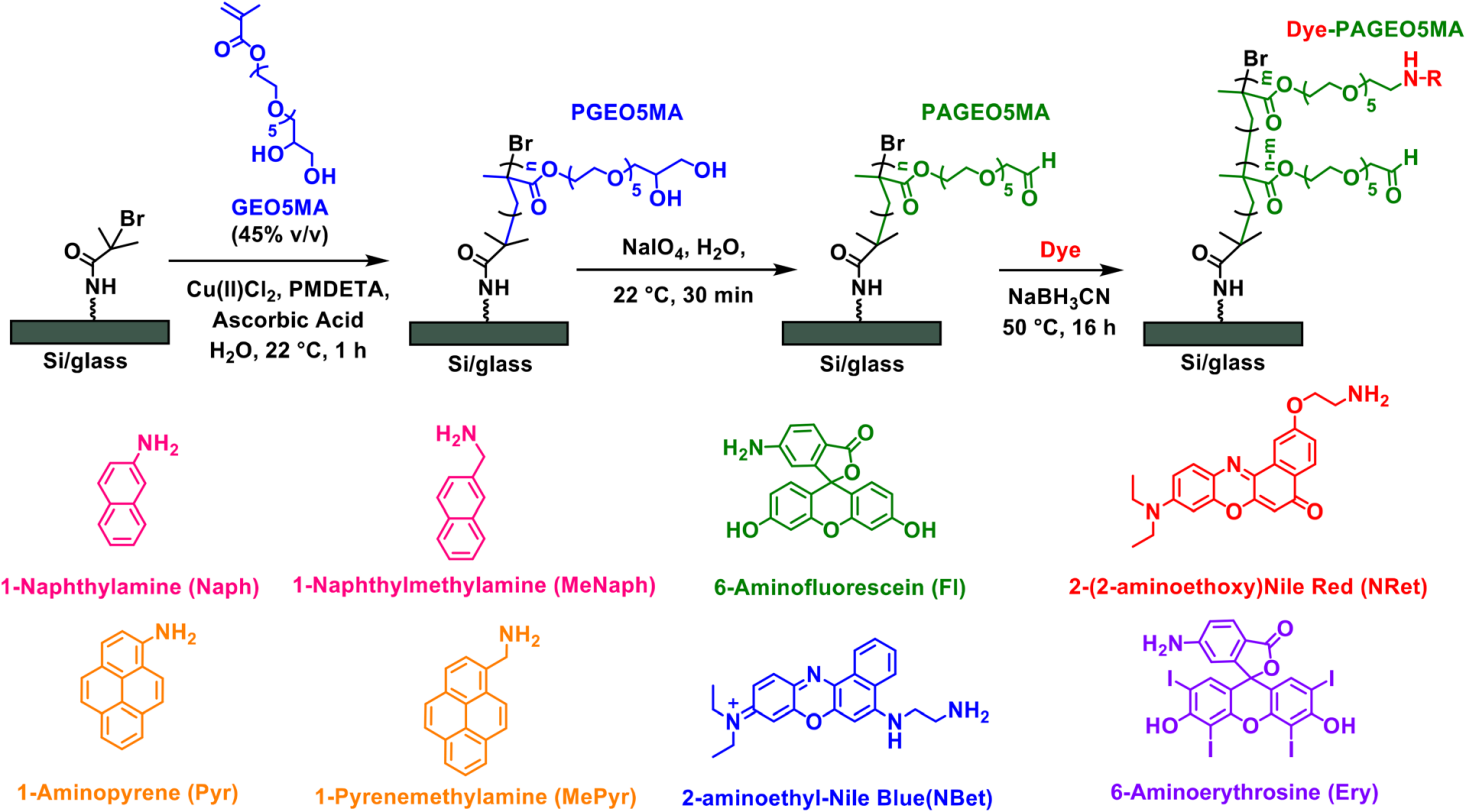
Growth of a PGEO5MA homopolymer brush from planar glass or a silicon wafer *via* SI-ARGET ATRP. Subsequent selective oxidation with sodium periodate under mild conditions produces the corresponding aldehyde-functional PAGEO5MA brush while conjugation with various amine-functional dyes produces a dye–PAGEO5MA pigment polymer antenna complex (PPAC).

Herein we report the conjugation of various dyes to surface-grafted PAGEO5MA layers *via* reductive amination (Scheme 1). We examine the effect of polymer grafting density, solvent quality, and dye size on the composition of the resulting PPAC. Using Nile Red as a probe, we characterize the photophysical properties of dye-loaded PAGEO5MA brushes using fluorescence lifetime imaging microscopy (FLIM). This technique enables the excited state lifetime for the PPAC to be compared with that recorded for spin-cast poly(methyl methacrylate) (PMMA) films containing free (non-conjugated) dye. Thus we address the following important question: does dye conjugation to a brush scaffold provide an effective means of minimizing dye–dye interactions and hence extending the excited state lifetime?

## Results and discussion

### Synthesis of polymer brush scaffolds with varying grafting densities

We hypothesized that the conjugation of large dye molecules to surface-grafted polymer scaffolds would be strongly influenced by steric constraints. Thus, to achieve high dye concentrations within such layers it is necessary to minimize steric repulsion. Variation of the polymer grafting density is known to influence the degree of steric repulsion between neighboring surface-grafted chains.^31^ Thus, we examined the polymerization kinetics and dye-conjugation chemistry for a range of grafting densities.

**Fig. 1:**
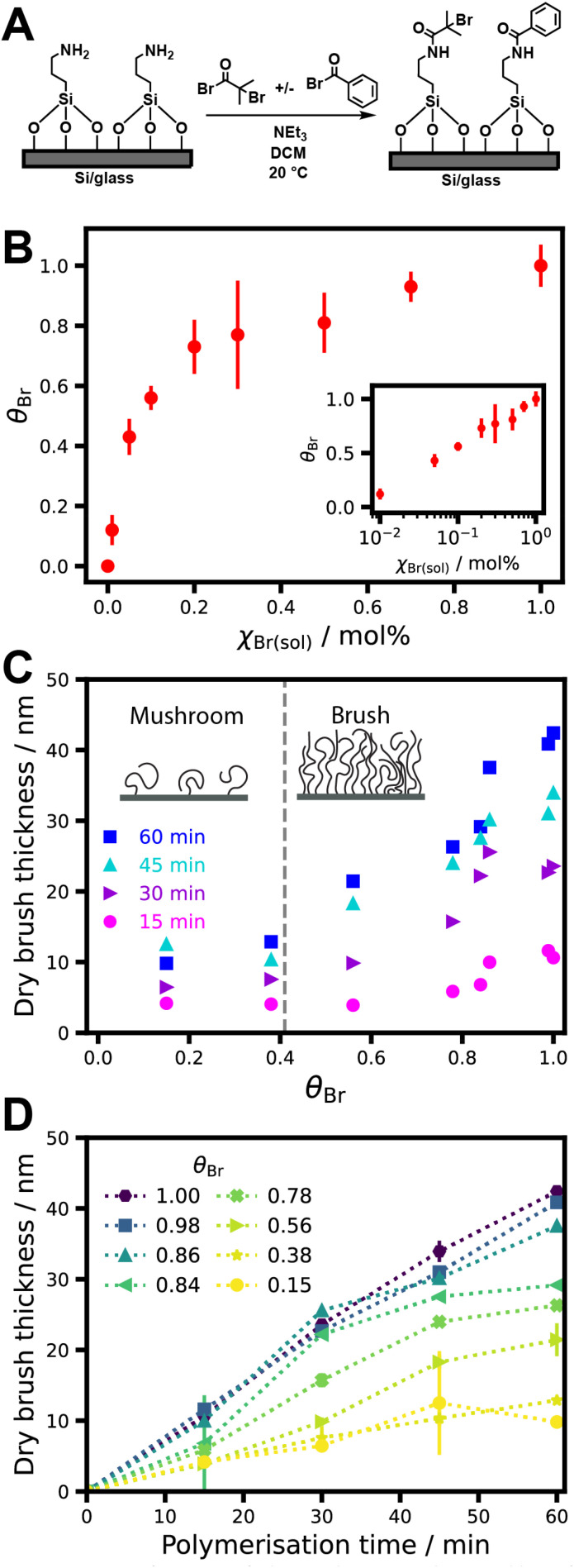
(A) Preparation of a series of planar substrates with a variable surface density of ATRP initiator groups. ((B) Fractional surface coverage of Br, *θ*_Br_, as a function of the mole fraction of BiBB in solution, *χ*_Br_, after reaction of aminosilane-functionalized wafers with a series of solutions containing various BiBB/BB binary mixtures. *θ*_Br_ is determined by normalizing the XPS Br 3d peak area by that observed for *χ*_Br_ = 1.0. (C) PAGEO5MA dry layer thickness against *θ*_Br_ as a function of polymerization time. The brush-to-mushroom transition is indicated by a vertical dashed line. (D) Ellipsometric dry brush thickness as a function of polymerization time for PAGEO5MA layers grown from silicon wafers at different *θ*_Br_.

Previously, we reported that PGEO5MA brushes bearing pendent *cis*-diol groups can be grown from initiator-functionalized silicon wafers at 22 °C using surface-initiated activators regenerated by electron transfer, atom transfer radical polymerization (SI-ARGET-ATRP).^24^ Linear growth kinetics were achieved over 2 h, indicating excellent control over the polymerization.^32^ In the present study, the surface concentration of ATRP initiator on a planar glass substrate or a silicon wafer was systematically varied by using an appropriate non-reactive diluent^33,34^ to control the brush grafting density for the PGEO5MA (and hence PAGEO5MA) chains. More specifically, an initial 3-aminopropyl siloxane (APTES) layer was modified by reaction with various binary mixtures of 2-bromoisobutyryl bromide (BiBB, initiator) and benzoyl bromide (BB, diluent) to yield a series of BiBB/BB–APTES functionalized surfaces (Fig. 1A). The initiator surface concentration on silicon wafers was determined using X-ray photoelectron spectroscopy (XPS). Fig. 1B shows the fractional surface coverage of Br, *θ*_Br_, as a function of the BiBB mole fraction, *χ*_Br_, in the BiBB/BB reaction mixture. *θ*_Br_ is calculated by normalizing the Br 3d XPS peak area with respect to the Br 3d peak area determined for an initiator layer prepared using BIBB alone, for which *χ*_Br_ = 1.0 (Fig. 1B). High resolution spectra are shown in Fig. S1. *θ*_Br_ increases rapidly at first as *χ*_Br_ increases, attaining a value of ∼0.7 at *χ*_Br_ = 0.2, but then changes more gradually as *χ*_Br_ approaches unity. If the binding constants for the two acyl halides were the same, the surface composition would simply reflect the solution composition. Thus the different behavior observed in Fig. 1B is attributed to a difference in the reactivity of BiBB and BB towards the APTES layer: aliphatic acyl halides are known to be more reactive than aromatic acyl halides.^35^ Thus *θ*_Br_ begins to approach unity at a lower *χ*_Br_ value than that expected if BiBB and BB had the same reactivity with the surface amine groups. The relationship between *θ*_Br_ and *χ*_Br_ was found to be logarithmic (see inset to Fig. 1B).^36^

Fig. 1C shows the mean thicknesses of dry PGEO5MA layers grown from silicon wafers for various *θ*_Br_ values, as determined by spectroscopic ellipsometry. The brush grafting density at *θ*_Br_ = 1.0 is assumed to be comparable to that for brushes prepared using a similar synthetic protocol.^31,37–39^ As *θ*_Br_ increases from 0.15 to 0.38, there is minimal change in the thickness of the dry surface-grafted layer. However, for *θ*_Br_ values above 0.4, the layer thickness increases as a linear function of *θ*_Br_ for longer polymerization times (>30 min). For *θ*_Br_ < 0.4, it is likely that the surface-grafted chains form a so-called mushroom layer, in which each chain has minimal interaction with its neighbors and the mean layer thickness is simply determined by the radius of gyration. However, when *θ*_Br_ exceeds 0.4, the surface-grafted chains adopt a brush conformation: increasing steric repulsion between neighboring chains leads to thicker layers at higher grafting densities.^31,40^ For shorter polymerization times (*e.g.* 15 min), the thickness of the surface-grafted layer only begins to increase significantly at higher *θ*_Br_, because the shorter chains are non-interacting at grafting densities that would otherwise yield brushes.

The following equation describes such behavior:^40^*σ* = (*hρN*_A_)/*M*_n_where *σ* is the grafting density, *h* is the brush thickness, *ρ* is the bulk density of the brush composition; *N*_A_ is Avogadro's number and *M*_n_ is the polymer molecular weight.

Brush growth kinetics observed for various initiator densities are shown in Fig. 1D. Linear growth kinetics are observed at the highest *θ*_Br_ values, suggesting limited termination of the propagating chains under such conditions. For intermediate *θ*_Br_ values (0.78–0.84) there is a deviation from linearity at longer polymerization times (2 h), which suggests an increase in termination at lower brush grafting densities. In principle, a reduction in grafting density should result in less chain termination because the probability of propagating radicals undergoing biomolecular termination is reduced.^41,42^ However, it has been reported that, for some polymer brushes grown at lower grafting densities, the higher chain mobility can increase the probability of solution-phase termination.^43^

### Synthesis of dye-functionalized surface-grafted PAGEO5MA

**Fig. 2:**
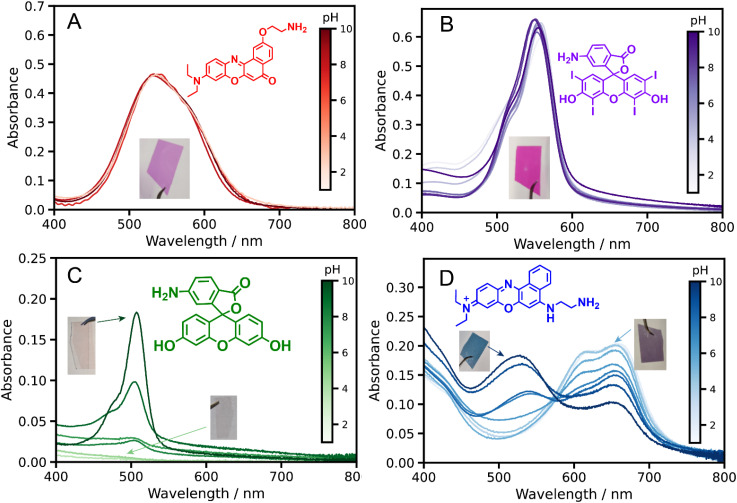
Visible absorption spectra recorded for dye-functionalized PAGEO5MA brushes grown from glass cover slips. Brushes were functionalized in turn with (A) Nile Red ethylamine (NRet), (B) 6-aminoerythrosine (Ery), (C) 6-aminofluorescein (Fl), and (D) Nile Blue ethylamine (NBet). All brushes were grown at the maximum grafting density and dyes were conjugated to methanol-swollen brushes. Spectra were recorded for dry brushes following their immersion in a series of aqueous solutions of varying pH.

The pendent *cis*-diol groups in surface-grafted PGEO5MA are efficiently converted into reactive aldehyde groups to yield PAGEO5MA *via* selective oxidation using a dilute aqueous solution of sodium periodate (NaIO_4_, 3.0 g mL^−1^) under mild conditions as previously reported.^24,25^ The resulting aldehyde-functional PAGEO5MA layers were then used as scaffolds to prepare PPACs. Four amine-functional dyes (NRet, Ery, Fl and NBet, see Scheme 1) were conjugated to the PAGEO5MA chains using reductive amination.^24^ A series of PAGEO5MA brushes (mean dry brush thickness ∼40 nm) were grown from glass coverslips before being immersed in a 5 µM methanolic solution of the dye containing a 1.5× molar excess of NaBH_3_CN relative to the dye. Brushes were allowed to react for 24 h at 50 °C before being rinsed in turn with copious methanol, ethanol and THF to remove any unreacted dye. In each case, the resulting dye-functionalized brushes were visibly colored (see inset photographs in Fig. 2). Attempts to prepare the same dye-functionalized brushes *via* Schiff base chemistry alone (*i.e.* solely *via* imine bond formation, with no *in situ* reductive amination) were unsuccessful: no dye was detected *via* either visible absorption spectroscopy or XPS (data not shown). This observation was attributed to the susceptibility of imines to hydrolysis, whereas reductive amination yields a stable, irreversible linkage enabling efficient conjugation of both aromatic and aliphatic amines to the PAGEO5MA brushes.^44–47^

Visible absorption spectra were recorded for dry dye-functionalized, fully dense PAGEO5MA brushes grown from glass coverslips. Fig. 2 shows representative spectra obtained for brushes derivatized using Nile Red ethylamine (NRet), 6-aminoerythrosine (Ery), 6-aminofluorescein (Fl) or Nile Blue ethylamine (NBet). Each dye-conjugated brush sample was immersed in aqueous solutions of varying pH, allowed to equilibrate for 5 min, and then dried under a stream of nitrogen gas before being transferred to the spectrophotometer.

An absorption maximum is observed at ∼528 nm for NRet-functionalized brushes along with a longer wavelength shoulder. The position of this absorption maximum does not change when varying the solution pH. For NRet in methanol (also shown in Fig. 2A) the absorption maximum is observed at 554 nm. The energy difference between the ground and excited states, S_0_ and S_1_ respectively, is reduced when using more polar solvents. Both states have large dipole moments, which leads to strong solvatochromism for this particular dye.^48–50^ Thus the blue-shifted absorption band observed for the corresponding NRet-conjugated brushes is attributed to the local environment of the dye within the swollen brush layer. For the sample shown in Fig. 2A, the dye concentration within the brush layer was estimated to be as high as 2.3 M using the Beer–Lambert law, with the path length taken to be the ellipsometric thickness of the dry brush layer.^51^ The effect of varying the dye concentration within the brush layer on the corresponding visible absorption spectrum recorded for the conjugated dye is investigated systematically below.

Visible absorption spectra obtained for 6-aminoerythrosine (Ery) functionalized PAGEO5MA brushes – for which the dye concentration within the brush layer was also estimated to be 2.3 M – were similar to those recorded for Ery in methanolic solution (Fig. S2). The aqueous solubility of the non-conjugated Ery dye is strongly dependent on the solution pH but its absorption maximum is pH-independent. However, the absorption spectrum recorded for Ery-functionalized PAGEO5MA brushes is much less pH-sensitive. This is attributed to the differing microenvironments for this dye in solution and within the brush layer. In particular, the effective dye concentration is much higher in the latter case.

PAGEO5MA brushes derivatized with Fl dye exhibited an absorption maximum at ∼510 nm after exposure to a weakly alkaline solution (pH 10). This band became progressively weaker on reducing the solution pH but with minimal change in its *λ*_max_. At pH 6, this band disappeared. This is consistent with the known behavior of fluorescein in solution, which displays only very weak absorbance under acidic conditions.^52^ On the other hand, an absorption is observed at ∼440 nm below pH 3 for an aqueous solution of non-conjugated Fl dye. This difference is attributed to the primary amine substituent in the non-conjugated dye. Such substituents are known to influence the pH-responsive behavior of fluorescein derivatives at low pH.^53^

The influence of solution pH is more dramatic for the absorption spectra recorded for NBet (Fig. 2D). At pH 10, a strong absorption band is observed at approximately 510 nm, along with a weaker band at 660 nm. On reducing the pH, the 510 nm band becomes progressively weaker and is no longer observed below pH 6, whereas the 660 nm feature becomes more intense. At around pH 6, a new shoulder appears at 610 nm. Both this feature and the 660 nm band become more intense below pH 6. For an aqueous solution of the free (non-conjugated) NBet dye at pH 10 (SI Fig. S2), a strong band is observed at approximately 660 nm along with a band at around 510 nm. On reducing the solution pH, the former band becomes stronger while the latter band becomes weaker until no longer being observed below pH 6. Thus, the pH-dependent behavior of NBet conjugated to a brush is broadly comparable to that for the same free dye in solution. However, one notable difference is that the 510 nm band is more intense than that at 660 nm for the NBet-conjugated brush, the latter feature is stronger for NRet in solution. Nile Red also displays strong solvatochromism^54,55^ so these differing relative band intensities at 510 and 660 nm are attributed to differences in the dye microenvironment.

### Influence of steric factors on dye conjugation to PAGEO5MA chains

It is well-established that steric repulsion between neighboring chains in the brush regime leads to chain stretching.^40^ In principle, optimization of dye-brush conjugation chemistry requires control over such steric interactions, which can be achieved by systematic variation of the brush grafting density while selecting dye molecules of differing size.^22^ Accordingly, three dyes of differing size, naphthylamine (Naph), NBet and Ery (see Scheme 1), were each conjugated in turn to PAGEO5MA chains grown using various grafting densities spanning the mushroom and brush regimes. The percentage of dye conjugated per methacrylic repeat unit was quantified by XPS. This mean degree of PAGEO5MA functionalization was calculated from N/O atomic ratios determined by XPS (see Fig. S3) using the method reported by Brotherton *et al.*^24^

**Fig. 3:**
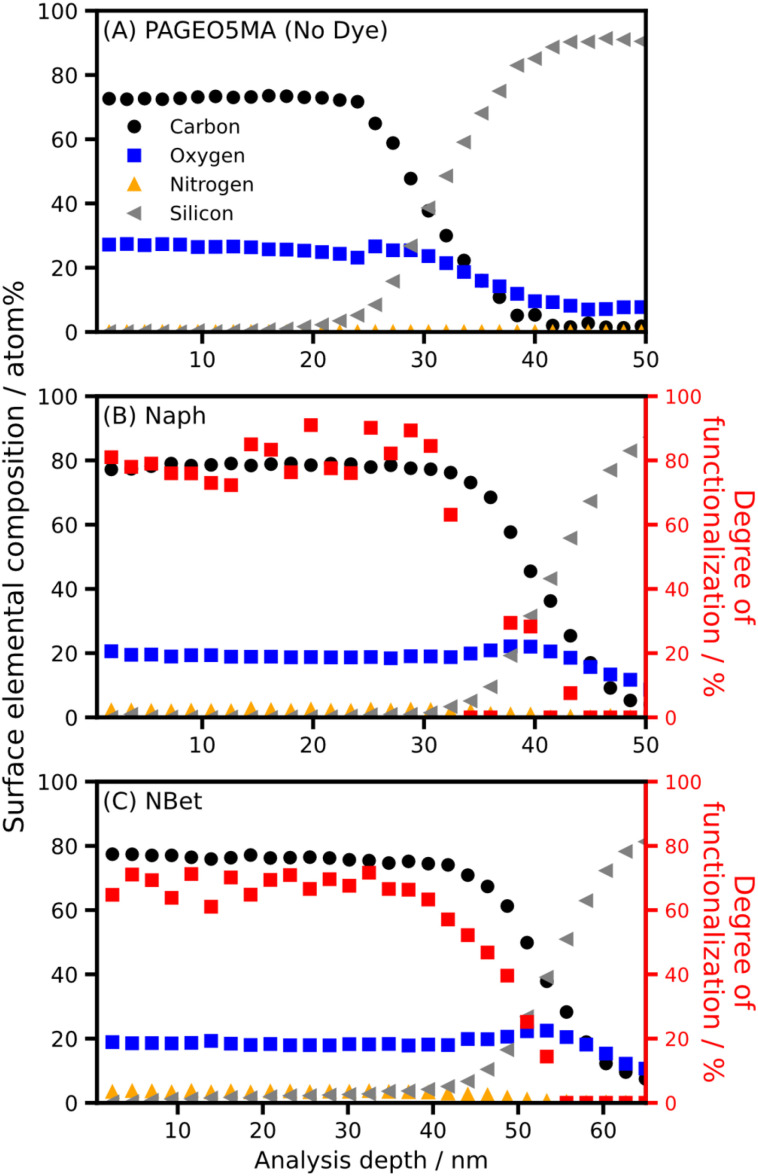
XPS depth-profile data for an unfunctionalized PAGEO5MA brush (A) and brushes conjugated to (B) Naph and (C) NBetN. Secondary axis (red) in B and C shows the calculated degree of functionalization (reactions performed in methanol).

To assess whether functionalization is confined to the near-surface region or occurs throughout the full brush thickness, XPS depth profiling measurements were performed for representative systems (Naph and NBet), as shown in Fig. 3. These data show a consistent signal associated with the dye-derived nitrogen species throughout the entire thickness of the dry brush, with no observable decrease in functional group concentration with increasing analysis depth until the polymer/substrate interface is approached (accompanied by rising silicon intensity). This indicates that dye incorporation is not limited to the outer surface but occurs uniformly throughout the film. These observations are consistent with previous reports on analogous systems functionalized using the same chemistry, which similarly demonstrate homogeneous modification throughout the brush thickness.^24^

Theoretical molecular volumes for Naph (58.1 Å^3^), NBet (163.6 Å^3^) and Ery (216.4 Å^3^) were calculated using density functional theory (DFT) at the M06-2X/def2-SVPD level of theory. The M06-2X functional is broadly validated for main-group organic molecules, including π-conjugated and noncovalently interacting systems typical of organic dyes, and is therefore appropriate for computing optimized structures and associated molecular properties.^56^ At the highest grafting density (*θ*_Br_ = 1.0), the degree of functionalization was found to be in the order Naph > NBet > Ery, which correlates inversely with the relative sizes of these three dyes (Ery > NBet > Naph). This is because inter-chain steric repulsion is sufficiently strong for such PAGEO5MA brushes that complete derivatization cannot be achieved for larger dyes.

**Fig. 4:**
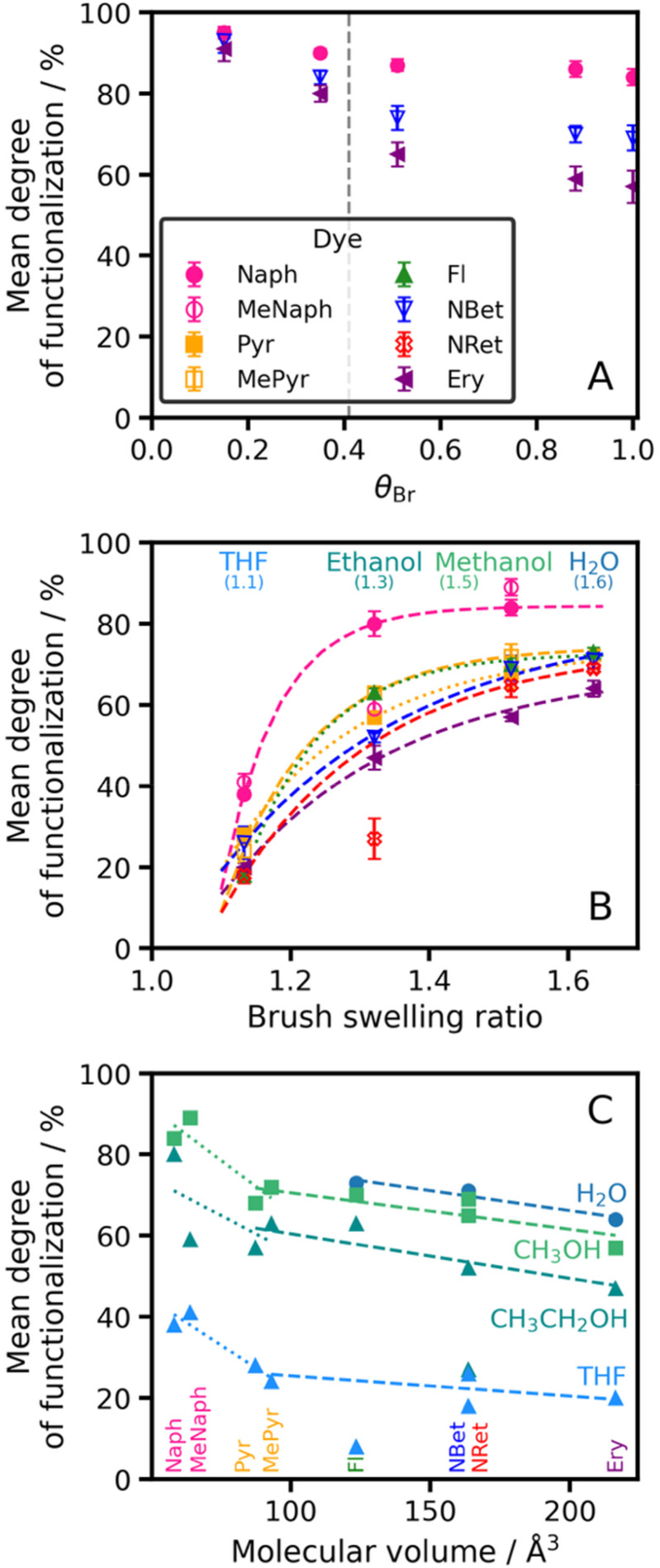
Factors affecting degree of dye conjugation for surface-grafted PAGEO5MA chains. (A) Variation in degree of functionalization with initiator fractional coverage, *θ*_Br_. The brush-to-mushroom transition is indicated by a dashed vertical line. (B) Variation in the mean degree of dye functionalization with the swelling ratio of the unmodified brush in four different solvents (*θ*_Br_ = 1.0 for all samples). Fitted lines were produced using half-sigmoidal fits. (C) Variation in degree of brush functionalization *vs.* theoretical dye molecular radius (*θ*_Br_ = 1.0 for all brushes, which were grown from glass slides). Dashed lines are a guide to the eye. Degrees of dye conjugation were determined by XPS from N 1s and O 1s signals for 8 different dyes (see legend in panel (A)). Reductive amination was conducted in methanol at a dye concentration of 5 µM.

However, higher degrees of dye conjugation could be achieved at lower chain grafting densities for a given dye (Fig. 4A). Within the brush regime (*θ*_Br_ > 0.4), there is only a modest increase in the degree of functionalization when reducing the grafting density. In contrast, the extent of dye functionalization increases significantly as *θ*_Br_ decreases within the mushroom regime (*θ*_Br_ < 0.4). Extrapolation suggests that, at a theoretical grafting density of zero, the degree of dye conjugation should be 100%. Indeed, for other reactive amines such derivatization can be achieved for free PAGEO5MA chains in aqueous solution.^25^ Clearly, the degree of dye conjugation for reactive surface-grafted PAGEO5MA chains can be modulated by adjusting the chain grafting density.^22,57^

The degree of swelling can be modulated by changing the solvent. To maximize the degree of dye conjugation, it is also necessary to select a good solvent for the dye of interest. The swelling ratio is defined as the solvated brush thickness divided by the dry brush thickness. Table 1 summarizes the swelling ratios obtained for fully-dense PAGEO5MA brushes immersed in four good solvents for the dyes studied herein: water, methanol, ethanol and THF. For comparison, data are also shown for a PGEO5MA brush, which is the *cis*-diol precursor to the aldehyde-functionalized PAGEO5MA brush (Scheme 1). The highest swelling ratios for both PGEO5MA and PAGEO5MA are achieved in water and these values are comparable to swelling ratios reported for other hydrophilic polymer brushes.^58–61^ When immersed in water, the more hydrophilic PGEO5MA brush exhibits a higher swelling ratio than the corresponding PAGEO5MA brush. As the solvent polarity decreases, concurrent with an increase in the size of the solvent molecules, both brushes become much less solvated, with minimal swelling observed for immersion in THF in each case.

**Table 1:** Summary of PGEO5MA and PAGEO5MA brush swelling ratios when immersed in four solvents (expressed relative to the corresponding dry brush in each case)

Solvent	Swelling ratio^a^		Solvent polarity^b^ (kcal mol^−1^)
	PGEO5MA	PAGEO5MA	
H_2_O	2.0 ± 0.1	1.6 ± 0.2	63.1
Methanol	1.7 ± 0.2	1.5 ± 0.1	55.5
Ethanol	1.3 ± 0.0	1.3 ± 0.0	51.9
THF	1.0 ± 0.1	1.1 ± 0.0	37.4

a Swelling ratios determined by spectroscopic ellipsometry for PGEO5MA and PAGEO5MA brushes with mean dry thicknesses of 40.1 nm and 36.6 nm, respectively. The brush swelling ratio is defined as the solvated brush thickness divided by the dry brush thickness.

b Dimroth–Reichardt solvent polarity values are used.^62^

Fig. 4B shows the mean degree of dye functionalization for fully-dense brushes (*θ*_Br_ = 1.0) determined by XPS after reaction in turn with each of the eight dyes shown in Scheme 1 when using THF, ethanol, methanol or water. On changing from THF (poor solvent) to water (good solvent), PAGEO5MA brushes become increasingly swollen. For each dye, a higher swelling ratio leads to a higher degree of dye conjugation. This is physically reasonable: if the brush is immersed in a good solvent, then the dye molecules can more readily penetrate the brush layer and hence react with a higher fraction of the pendent aldehyde groups within it. These observations are consistent with previous studies of the derivatization of poly(2-(dimethylamino ethyl)methacrylate) brushes.^63^ This is because it is easier for this dye to access the aldehyde functional groups when the PAGEO5MA chains adopt a highly stretched conformation. Moreover, the data in Fig. 4A also show that higher degrees of dye conjugation are achieved at lower chain grafting densities. Within the mushroom regime the collapsed PAGEO5MA chains are well-separated from each other so the steric barrier to dye ingress is minimal. However, the lower chain grafting density means that the overall local dye concentration that can be achieved within such relatively diffuse surface layers is significantly lower than that for a fully dense brush layer.

Finally, the relationship between dye size and the degree of dye conjugation was examined (Fig. 4C). Fig. 4C shows the relationship between the degree of dye functionalization for surface-grafted aldehyde-functionalized polymer chains in THF, ethanol, methanol or water and the theoretical dye volume (calculated using DFT).^56^ Clearly, using larger dyes (>80 Å^3^) leads to lower degrees of dye functionalization. This is attributed to the greater steric congestion.

In summary, reductive amination is an efficient method for the attachment of various amine dyes to surface-grafted aldehyde-functionalized polymer chains. High degrees of dye conjugation can be achieved, with the mole fraction of dye-conjugated methacrylic repeat units approaching unity under optimized conditions. For a given chain grafting density, maximizing the solvation of the polymer chains by selecting an appropriate solvent leads to a higher degree of dye functionalization.

### Absorption spectra for Nile Red-functionalized PAGEO5MA brushes

The main aim of this study was to construct PPACs comprising dye-functionalized surface-grafted polymer scaffolds. To achieve the desired exciton dynamics, such PPACs must offer a sufficiently high dye concentration combined with good control over the spatial location of dye molecules within the polymer layer. It is particularly important to minimize dye–dye interactions, which would otherwise cause exciton quenching. Hence we investigated the spectroscopic properties of dye-functionalized brushes in detail, selecting NRet as a model dye. Nile Red has been widely used in cell biology as a solvatochromic membrane-selective stain:^64^ its absorption and fluorescence emission wavelength each depend on the local dye environment.^48^

**Fig. 5:**
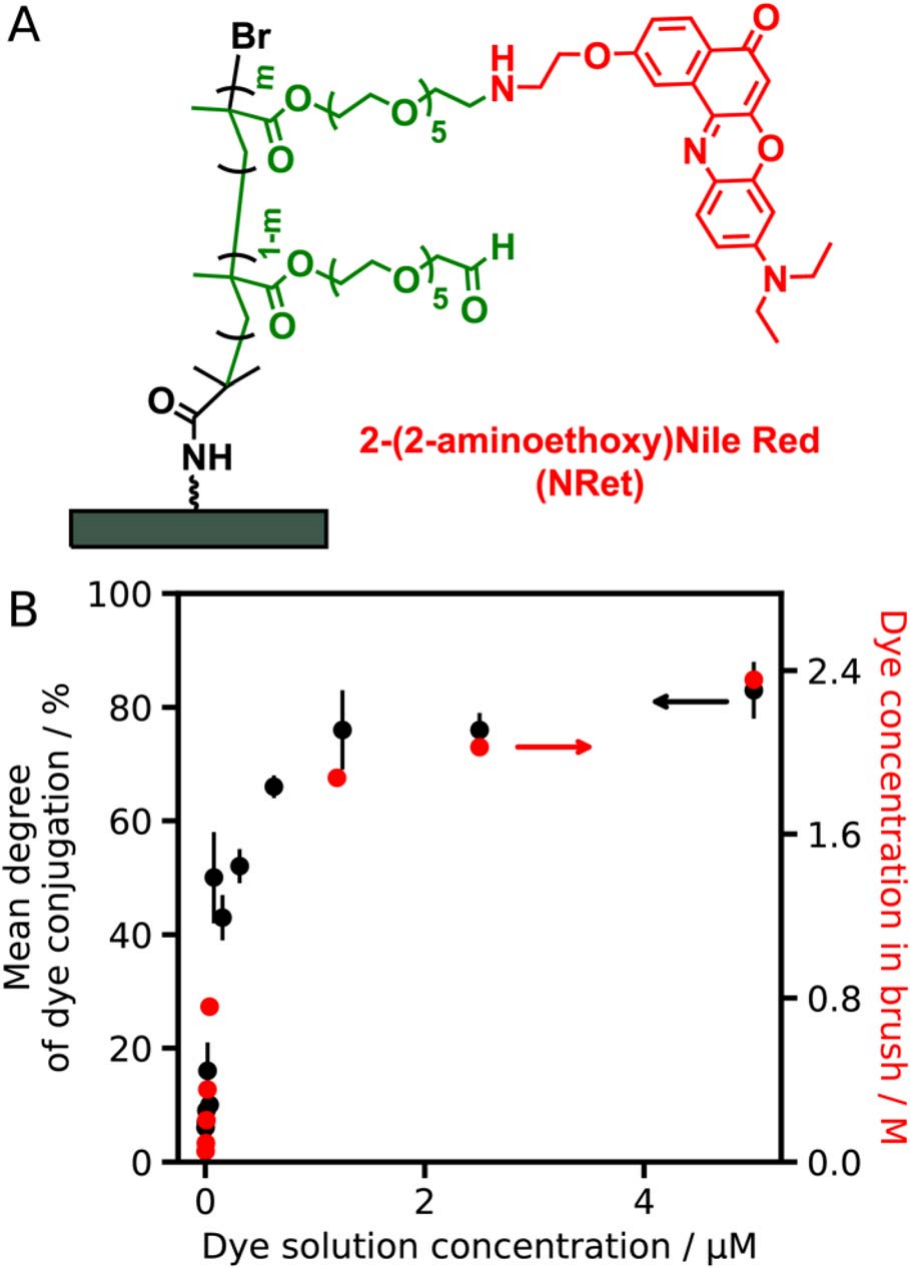
(A) Chemical structure of a P(NRet)AGEO5MA PPAC. (B) Variation in the mean degree of functionalization (black data points) and local dye concentration within a fully dense PAGEO5MA brush layer (red data points) depending on the NRet solution concentration. The mean degree of dye conjugation was determined *via* XPS (see main text for further details).

NRet was conjugated to PAGEO5MA brushes by reductive amination (Fig. 5A) using a series of methanolic dye solutions of varying concentration [*N.B.* NRet is insoluble in water]. The mean degree of dye conjugation was determined *via* XPS as a function of NRet concentration in solution (Fig. 5B, black data points). These data are consistent with first-order binding kinetics: a linear relationship is observed when the data are replotted on a logarithmic axis, see Fig. S4. The dye concentration within each brush was determined from visible absorption spectra recorded for dry brushes by applying the Beer–Lambert law, where the dry thickness of the dye-conjugated brush (∼40 nm) is the path length and the molar extinction coefficient of the conjugated dye was taken to be that of Nile Red (38 000 M^−1^ cm^−1^).^65^

To determine whether the dye was distributed uniformly throughout the functionalized brushes, samples were characterized using spectroscopic ellipsometry and the data were modeled using both uniform and graded optical profiles in both the dry state and after equilibration in pH 9 (Fig. S5). It was found that the uniform layer model provided the most robust and statistically justified representation of the SE data across all samples and conditions investigated.

**Fig. 6:**
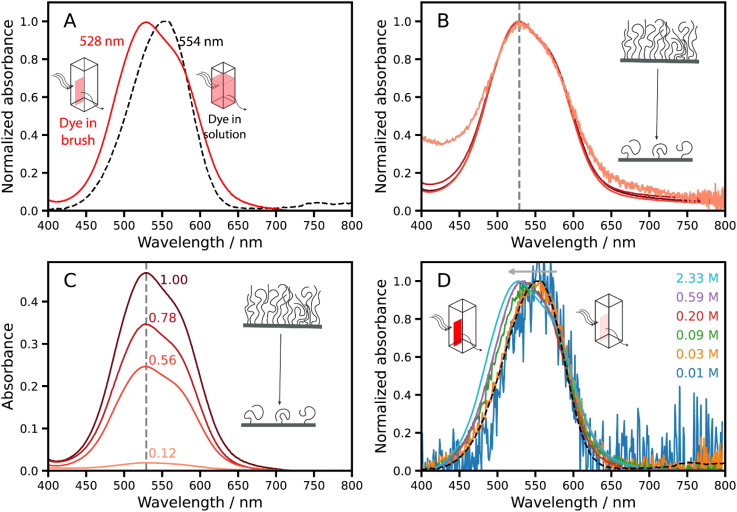
Steady state absorbance spectra recorded for Nile Red functionalized brushes. (A) Comparison of spectra recorded for NRet dissolved in 5 µM methanol solution (black dashed line) and for a dry NRet-functionalized PAGEO5MA brush grown from a glass substrate. This sample has the highest brush grafting density and the highest local dye concentration within the brush (2.33 M). (B) Absorbance and (C) normalized absorbance recorded for an NRet-functionalized PAGEO5MA brush prepared using a series of initiator fractional coverages, *θ*_Br_. The highest grafting density brush has a concentration of 2.33 M. Cartoon inset illustrates the change in brush density. (D) Selected normalized absorbance spectra recorded for a series of NRet-functionalized PAGEO5MA brushes containing various dye concentrations. Absolute intensity data obtained for this dataset are shown in Fig. S7. The horizontal grey arrow shown in (D) indicates the blue shift in the maximum wavelength with increasing dye concentration. Dashed black line indicates the normalized spectra for NRet molecularly dissolved in methanol.

The visible absorption spectrum recorded for free NRet dye dissolved in methanol is shown in Fig. 6A (black dashed spectrum). This spectrum has an absorption maximum at 554 nm and is identical to that reported for a commercially available, non-aminated Nile Red dye dissolved in methanol.^66^Fig. 6A also shows the absorption spectrum recorded for a NRet-functionalized brush prepared by immersing a fully-dense PAGEO5MA brush into a 5 µM methanolic solution of NRet (red spectrum). The concentration of conjugated dye molecules within this brush layer is estimated to be 2.33 M. The absorption maximum for NRet-functionalized brushes at this concentration is observed at around 528 nm and there is also a discernible shoulder at approximately 580–590 nm. Nile Red exhibits strong solvatochromism because the energy difference between its ground and excited states is reduced in the presence of more polar solvents.^48^ Thus the blue-shifted absorption observed for the corresponding dye-functionalized brush could reflect a subtle change in the local environment for this chromophore.

To examine whether excitonic coupling between neighboring chains in the brush layer might occur, a series of brushes were grown with various grafting densities. Fig. 6B shows normalized spectra recorded for the same dye-functionalized brush system prepared with *θ*_Br_ ranging from 0.12 to 1.00. The absolute intensities of the absorption maxima of these films (Fig. 6C) depend on the grafting density, because the total amount of material within the sampled area also varies with the grafting density. However, there is no other significant change in the spectra as the grafting density is varied, indicating that excitonic coupling between surface-grafted chains is weak.

To further explore whether the difference between the brush and solution spectra shown in Fig. 6A was attributable to the local dye concentration, measurements were conducted on a series of fully-dense dye-functionalized brushes prepared using a wide range of NRet concentrations (Fig. 6D). At the lowest dye concentration of 0.01 M, the absorption spectrum obtained for the NRet-functionalized brush is essentially identical to that of the free dye in solution. For higher NRet concentrations within the brush layer, the absorption maximum becomes slightly blue-shifted. For dye concentrations of 0.20 to 2.33 M, the spectra become increasingly asymmetric because this absorption band is sensitive to the local dye environment and potentially to aggregation.^48^

**Fig. 7:**
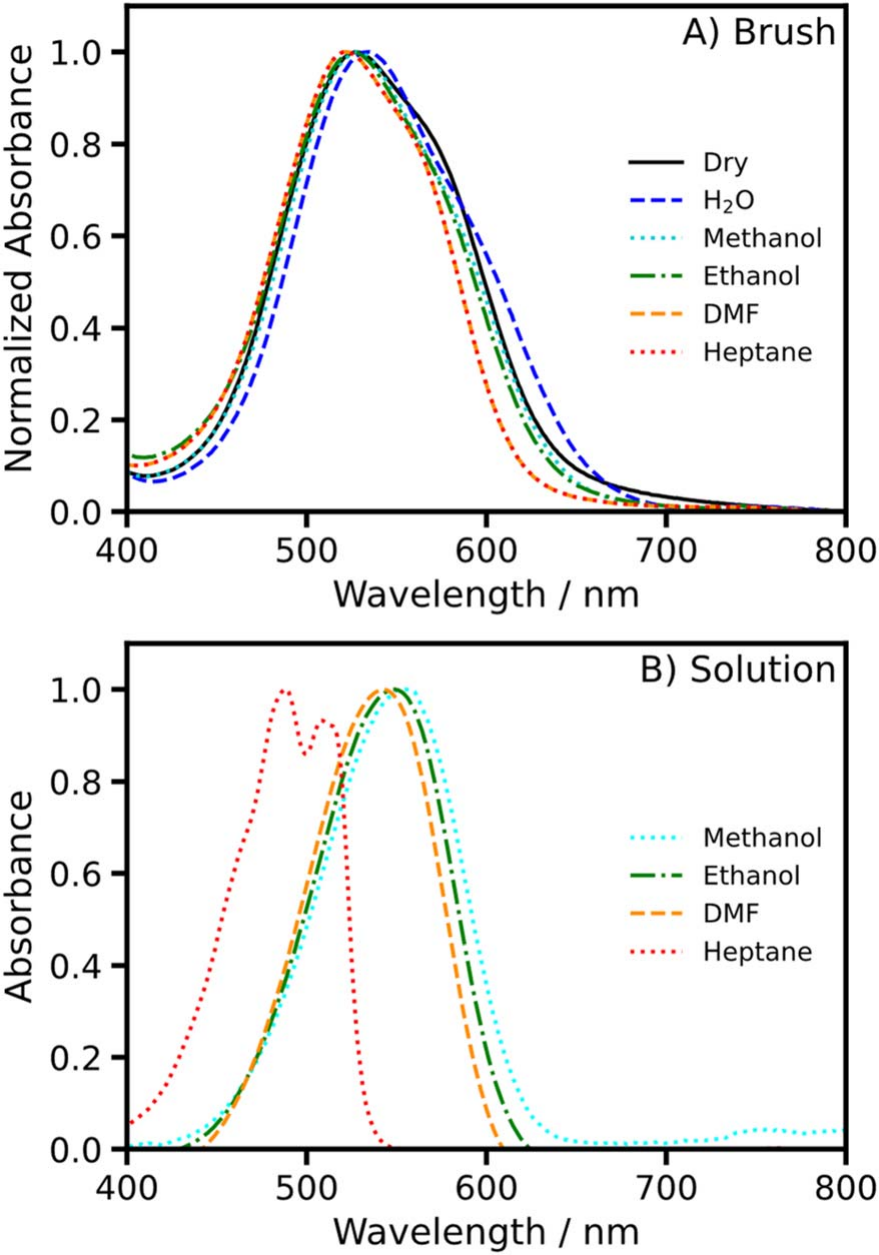
(A) Absorption spectra recorded for an NRet-functionalized brush immersed in various solvents. Brushes were prepared at the highest achievable grafting densities and reacted with a 5 µM NRet solution to target the maximum dye loading. (B) Shows the absorption spectra recorded for free (non-conjugated) NRet dissolved in the same solvents.

Fig. 7A shows absorption spectra recorded for fully-dense NRet-functionalized brushes containing the highest observed NRet concentration (2.33 M), both in the dry state and after their immersion in five different solvents. The absorption spectrum recorded for Nile Red dissolved in *n*-heptane differs significantly from that in methanol (Fig. 7B), which is consistent with literature reports.^48^ In contrast, spectra recorded for NRet-functionalized brushes immersed in *n*-heptane (non-swollen collapsed brush) and in methanol (highly swollen brush) are almost indistinguishable, suggesting that the local dye environment is mainly determined by the dielectric properties of the polymer chains, rather than the choice of solvent.

### Fluorescence emission spectra and excited state lifetime measurements

**Fig. 8:**
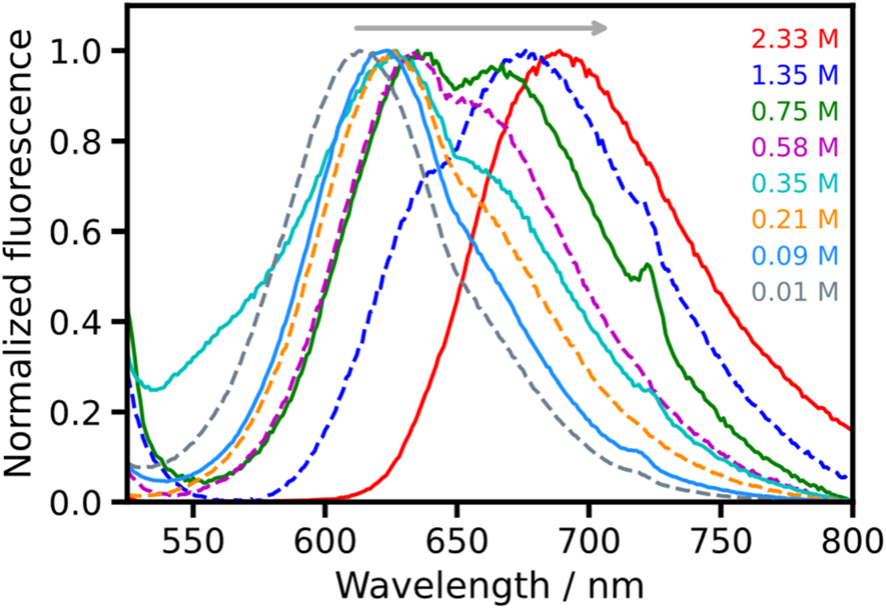
Normalized fluorescence spectra recorded for NRet-functionalized PAGEO5MA brushes prepared at various dye loadings. The excitation wavelength was 480 nm. Spectra for samples prepared with additional dye loadings are shown in Fig. S8.

Fluorescence emission spectra were acquired for NRet-functionalized brushes containing various dye loadings using a 480 nm excitation source (Fig. 8; an extended data set is also provided in Fig. S8 in the SI). At the lowest dye loadings of 10–210 mM, these spectra show a strong emission band at 613 nm with a weak shoulder at ∼655 nm. Higher dye concentrations produce red-shifted spectra with this shoulder becoming more prominent. At 0.75 M NRet, this feature is almost as intense as the main band. Above 0.75 M, the latter feature becomes weaker and, when the NRet concentration within the brush is approximately 2.33 M, a single absorption band is again observed, albeit at a significantly higher wavelength (689 nm).

At the highest NRet concentrations shown in Fig. 8, dye–dye interactions may occur within the brush layer. Thus, one possible explanation for the change in the emission spectra recorded for NRet conjugated to PAGEO5MA brushes is aggregation-induced dual fluorescence owing to the existence of two emitting states, monomeric NRet (higher energy) and NRet aggregates (lower energy).^67^ However, it is also feasible that these spectral changes simply reflect a change in the dielectric properties of the brush layer at higher dye concentrations and/or that self-absorption effects are present. In this context, Gajo *et al.* reported a detailed analysis of fluorescence emission spectra for free Nile Red dissolved in a series of solvents with a range of dielectric constants.^48^ Importantly, the spectral changes shown in Fig. 8 lie within the range of variation described in this prior study. Thus it may not be necessary to invoke dye–dye interactions to explain such spectroscopic observations.

Nevertheless, for brush layers that contain the highest dye concentrations, dye aggregation remains a possibility. Hence we employed fluorescence lifetime imaging microscopy (FLIM)^68^ to test this hypothesis. Dye aggregation often quenches the excited state leading to a reduction in its lifetime. The effective cross-sectional area of surface-grafted chains is ∼2 nm^31^ and, on average, there are estimated to be ∼4 repeat units per nm along a polymer chain (assuming no chain-folding for the fully-extended brush). Thus, for dye concentrations below 0.21 M within a fully-dense brush layer (which is equivalent to a degree of brush functionalization of ∼10%) and a stochastic distribution of dye, we estimate that the mean separation distance between dye molecules along the polymer chain should be less than mean spacing between surface-grafted chains. Hence the excited state lifetime should be influenced by the probability of excitation transfer between dye molecules on nearest-neighbor chains, which will decrease with dye concentration until it becomes vanishingly small. However, for dye concentrations of 0.35 M or above (degree of dye functionalization ≥20%), excitation transfer between neighboring dye molecules conjugated to the same polymer chain should become increasingly likely. Thus the excited state lifetime should be sensitive to the rates of both intra-chain and inter-chain excitation transfer within this dye concentration regime.

Excited state lifetimes were determined for a series of such dye-functionalized brushes by measuring time-resolved fluorescence using time correlated single photon counting (TCSPC). Measurements were made at 100 different locations on each brush sample. The resulting decay curves can be either analyzed individually or averaged, enabling the spatial distribution of the NRet dye within the brush layer to be assessed. The mean fluorescence lifetime *τ*_mean_ was calculated as the amplitude-weighted mean of the lifetimes associated with a minimum of 100 individual decay curves:1
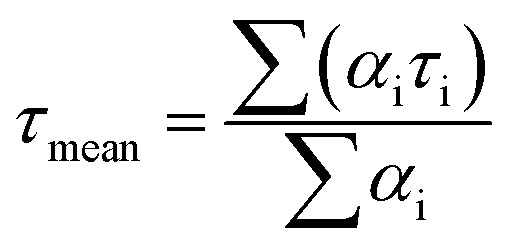
where *α*_i_ and *τ*_i_ are the fitted amplitudes and the corresponding lifetimes of each exponential component.

**Fig. 9:**
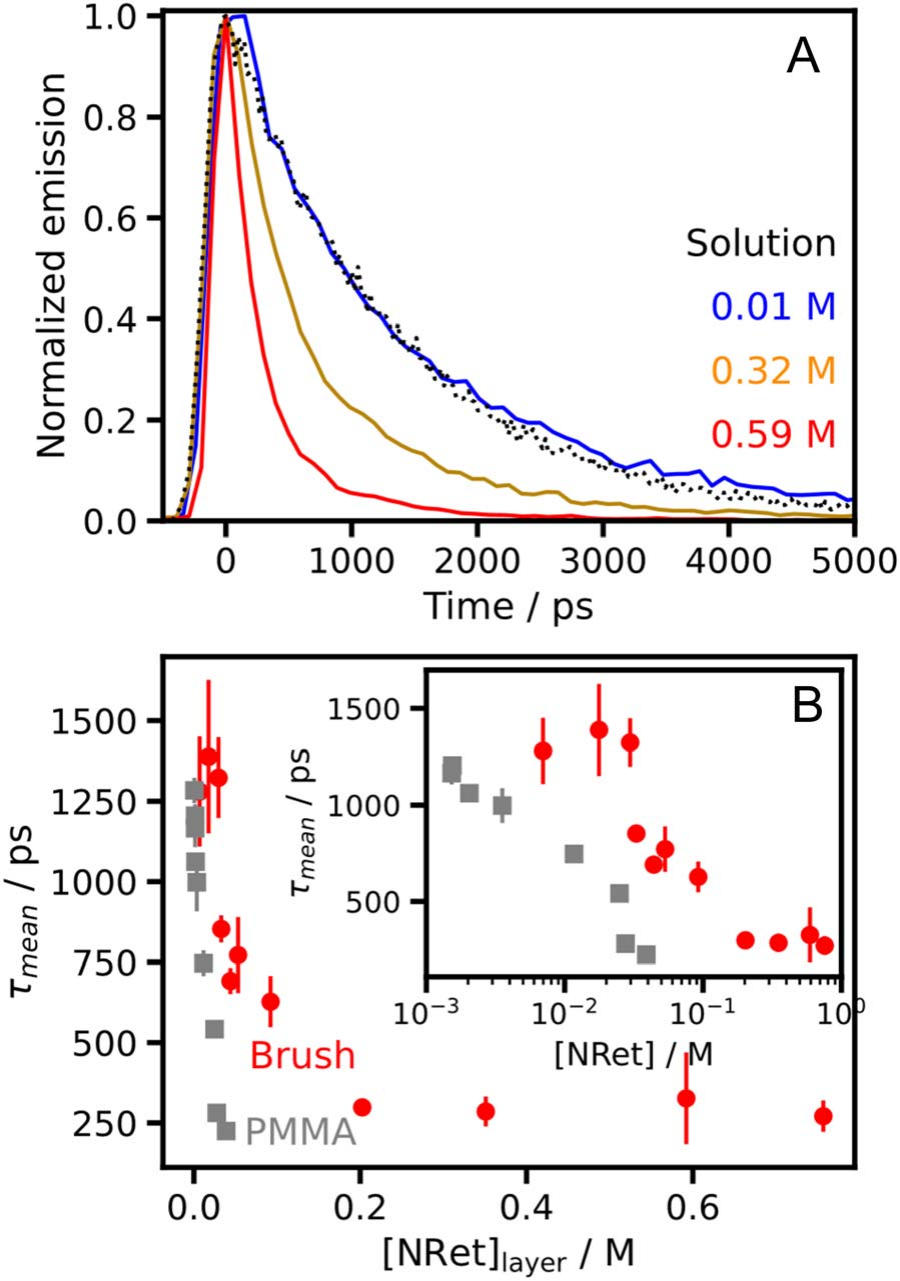
(A) Representative fluorescence decay curves (colored lines) recorded for NRet-functionalized brushes at different dye concentrations. Measurements were recorded at 100 locations across the surface of each sample. A decay curve is also shown for a 4.6 µM solution of the unconjugated dye in methanol (black dots). (B) Variation in the mean excited state lifetime of NRet with dye concentration for both this dye/brush system and for free Nile Red dispersed within PMMA spin-cast films. Data are averaged over 100 spectra with error bars shown as the standard deviation.

Fig. 9A shows fluorescence decay data recorded first for NRet in solution (dotted black line) and then after its conjugation to fully dense brushes at various dye concentrations. In each case the decay curve is the mean of 100 measurements. For a brush layer containing 0.01 M NRet, the fluorescence intensity decays at a similar rate to that recorded for the free dye in methanolic solution. In this case, *τ*_mean_ was determined to be 1.3 ± 0.1 ns. However, a faster rate of fluorescence decay is obtained for higher NRet concentrations. Accordingly, *τ*_mean_ was determined for a larger library of brushes over a wide range of NRet concentrations.

Fig. 9B shows the dependence of *τ*_mean_ on the NRet concentration within the brush layer. At the lowest dye concentrations (<0.04 M), the mean excited state lifetime measured at 100 different locations is 1.3 ± 0.1 ns. This value is comparable to that observed for the free dye dissolved in methanol (see Fig. 9A), as well as for Nile Red solubilized within membranes and vesicles.^69^ This lifetime is independent of the dye loading for dye concentrations ≤0.04 M, which suggests that both inter-chain and intra-chain excitation transfer are effectively controlled by the brush layer. Progressively shorter lifetimes are observed above this critical concentration, reaching a minimum value of ∼240 ps at an NRet concentration of 1 M. Between 0.03 and 0.21 M dye, there is a progressively higher probability of inter-chain dye–dye excitation transfer, which leads to a concomitant reduction in the excited state lifetime. Above a dye concentration of 0.21 M dye, there is also a higher probability of intramolecular excitation transfer, which leads to a faster rate of excited state quenching. These observations are consistent with the data in Fig. 8, which show that the absorption spectrum begins to change significantly at concentrations above 0.21 M, and with our estimate that above this concentration the mean dye–dye separation along the polymer chain is shorter than the mean separation between neighbouring surface-grafted chains.

To understand the significance of the data in Fig. 9B, we also measured the excited state lifetime for free Nile Red dye dispersed within poly(methyl methacrylate) (PMMA) films prepared by spin-casting, which is widely employed for fabricating thin uniform films of molecular dyes within a transparent polymeric matrix.^70^Fig. 9B shows such fluorescence data: the excited state lifetime for free Nile Red falls rapidly, reaching ∼200 ps at a dye concentration of 0.03 M. In striking contrast, the excited state lifetime remains unchanged at ∼1300 ps for the NRet-functionalized brush at the same dye concentration, and decreases much more slowly as the dye concentration within the layer increases. The reduction in the excited state lifetime observed for spun-cast PMMA films prepared using a dye concentration of up to 0.09 M is attributed to aggregation of the non-conjugated dye molecules during film formation. For this dye concentration regime, it is clear that dye conjugation to brush chains is a very effective mechanism for minimizing such aggregation. For higher degrees of brush functionalization, lower excited state lifetimes are observed. However, the excited state lifetime always exceeds 200 ps (*i.e.* the lifetime measured for non-conjugated Nile Red dye within the spun-cast PMMA films) up to a dye concentration of 0.21 M, which is seven times higher than the dye concentration of the free dye within the spun-cast PMMA film.

In summary, dye conjugation to PAGEO5MA brush chains does not eliminate aggregation across the entire concentration range. Nevertheless, this strategy provides effective control of dye aggregation at low dye concentrations and significantly reduces the degree of dye aggregation up to dye concentrations of 0.21 M. This is a highly significant improvement in performance compared to the corresponding dye-loaded spin-cast PMMA films and has important implications for the construction of next-generation PPCs.

Pigment–protein photosynthetic light-harvesting complexes provide an attractive paradigm for the design of molecular photonic materials: pigment molecules (chlorophylls and carotenoids) are organized precisely in 3D by peptide scaffolds, conferring good control over exciton dynamics. For example, LHCs achieve high concentrations of excitons, without quenching of excited states. Our aim in this work was to design synthetic scaffolds capable of offering comparable performance to that of photosynthetic LHCs. The data presented herein demonstrate that conjugation of dye molecules to polymer brushes enables effective control over dye aggregation. This approach yields significantly longer excited state lifetimes compared to those observed for free dye dispersed within spun-cast films and enables higher useful dye loadings to be achieved. However, the excited state lifetimes are still reduced within such PPACs at very high dye concentrations (∼0.3 M), which suggests that dye aggregation is not completely eliminated. Thus further refinements will be required before such brush layers can fully replicate the exquisite control over exciton dynamics observed for LHCs.

## Conclusions

We have developed a biologically-inspired approach for the design of next-generation photonic materials, in which programmable pigment–polymer complexes (PPACs) are produced *via* conjugation of various amine-functionalized dyes to an aldehyde-functionalized PAGEO5MA brush. Dye conjugation to a brush scaffold provides an effective means of minimizing dye–dye interactions and hence extending the excited state lifetime. The fraction of dye-functionalized repeat units, *x*_dye_, is controllable and approaches unity under optimum conditions.

Steric congestion plays an important role in regulating dye conjugation to the brush chains; control over this parameter provides a high degree of programmability for the resulting PPCs. More specifically, a higher degree of dye conjugation is favored by using a highly swollen brush, reducing the brush grafting density, and minimizing the size of the dye molecule. Changes in absorption spectra recorded for brushes before and after their functionalization with Nile Red ethylamine (NRet) are attributable to the modified dielectric environment within the brush layer. Concentration-dependent fluorescence emission spectra are ascribed to variations in the dielectric properties of the brush layer owing to high degrees of dye conjugation.

After binding to a PAGEO5MA brush at a dye concentration of no more than 0.04 M, the excited state lifetime for NRet in such a PPC is 1.3 ± 0.1 ns, which is comparable to that observed for a dilute solution of this dye in methanol (1.17 ± 0.01 ns). Importantly, this excited state lifetime is significantly longer than that observed for spin-cast films containing non-conjugated Nile Red at the same dye concentration (∼0.2 ns), which is attributed to minimal interactions between neighboring conjugated dye molecules within the brush layer. In summary, efficient dye conjugation chemistry confers good control over exciton dynamics in dye-loaded polymer brushes relative to that achieved for spin-cast films containing the same non-conjugated dye molecules. Such PPCs offer a new and potentially versatile route to the production of programmable photonic materials.

## Author contributions

ECJ and DB prepared dye-functionalised polymer brushes, ECJ carried out measurements of brush growth, UV-vis measurements, spectroscopic ellipsometry measurements and modelling of brush swelling, TY carried out FLIM measurements and modelled data, KG carried out density functional modelling of dye molecules, JW carried out modelling of ellipsometric data, DBH carried out XPS measurements, TY and JPP prepared spin-cast films of dye molecules, ECJ, SPA and GJL drafted the manuscript and all other authors edited it.

## Conflicts of interest

There are no conflicts to declare.

## Supplementary Material

SC-OLF-D6SC01202G-s001

## Data Availability

Data for this paper are available at the University of Sheffield's Online Data Archive (ORDA) at https://figshare.com/s/62e45dbc654d618dcc3e. Supplementary information (SI): materials and characterization techniques, synthetic protocols, characterization data for NBet, high resolution XPS spectra recorded for BiBB/BB functionalized silicon wafers and PAGEO5MA brushes conjugated to a range of amine-functional dyes. Absorbance spectra recorded for 6-aminoerythrosine, 6-aminofluorescein and NBet in solution. Unnormalized absorbance spectra recorded for P(NRet)AGEO5MA brushes and emission data for the same films recorded over an extended range of concentrations. See DOI: https://doi.org/10.1039/d6sc01202g.

## References

[cit1] Hedley G. J., Ruseckas A., Samuel I. D. (2017). Light Harvesting for Organic Photovoltaics. Chem. Rev..

[cit2] Spano F. C., Silva C. H. (2014). J-Aggregate Behavior in Polymeric Semiconductors. Annu. Rev. Phys. Chem..

[cit3] Bardeen C. J. (2014). The Structure and Dynamics of Molecular Excitons. Annu. Rev. Phys. Chem..

[cit4] Bronstein H., Nielsen C. B., Schroeder B. C., McCulloch I. (2020). The Role of Chemical Design in the Performance of Organic Semiconductors. Nat. Rev. Chem..

[cit5] Coropceanu V., Cornil J., da Silva Filho D. A., Olivier Y., Silbey R., Brédas J.-L. (2007). Charge Transport in Organic Semiconductors. Chem. Rev..

[cit6] Rider M. S., Johnson E. C., Bates D., Wardley W. P., Gordon R. H., Oliver R. D., Armes S. P., Leggett G. J., Barnes W. L. (2024). Strong Coupling in Molecular Systems: A Simple Predictor Employing Routine Optical Measurements. Nanophotonics.

[cit7] Blankenship R. E. , Molecular Mechanisms of Photosynthesis, Wiley-Blackwell, 2nd edn, 2014

[cit8] Şener M. K., Olsen J. D., Hunter C. N., Schulten K. (2007). Atomic-Level Structural and Functional Model of a Bacterial Photosynthetic Membrane Vesicle. Proc. Natl. Acad. Sci. U. S. A..

[cit9] Scholes G. D., Fleming G. R., Chen L. X., Aspuru-Guzik A., Buchleitner A., Coker D. F., Engel G. S., van Grondelle R., Ishizaki A., Jonas D. M., Lundeen J. S., McCusker J. K., Mukamel S., Ogilvie J. P., Olaya-Castro A., Ratner M. A., Spano F. C., Whaley K. B., Zhu X. (2017). Using Coherence to Enhance Function in Chemical and Biophysical Systems. Nature.

[cit10] Duan H.-G., Prokhorenko V. I., Cogdell R. J., Ashraf K., Stevens A. L., Thorwart M., Miller R. J. D. (2017). Nature Does Not Rely on Long-Lived Electronic Quantum Coherence for Photosynthetic Energy Transfer. Proc. Natl. Acad. Sci. U. S. A..

[cit11] Lishchuk A., Csányi E., Darroch B., Wilson C., Nabok A., Leggett G. J. (2022). Active Control of Strong Plasmon–Exciton Coupling in Biomimetic Pigment–Polymer Antenna Complexes Grown by Surface-Initiated Polymerisation from Gold Nanostructures. Chem. Sci..

[cit12] Csányi E., Hammond D. B., Bower B., Johnson E. C., Lishchuk A., Armes S. P., Dong Z., Leggett G. J. (2024). XPS Depth-Profiling Studies of Chlorophyll Binding to Poly(Cysteine Methacrylate) Scaffolds in Pigment–Polymer Antenna Complexes Using a Gas Cluster Ion Source. Langmuir.

[cit13] Tsargorodska A., Cartron M. L., Vasilev C., Kodali G., Mass O. A., Baumberg J. J., Dutton P. L., Hunter C. N., Törmä P., Leggett G. J. (2016). Strong Coupling of Localized Surface Plasmons to Excitons in Light-Harvesting Complexes. Nano Lett..

[cit14] Lishchuk A., Kodali G., Mancini J. A., Broadbent M., Darroch B., Mass O. A., Nabok A., Dutton P. L., Hunter C. N., Törmä P., Leggett G. J. (2018). A Synthetic Biological Quantum Optical System. Nanoscale.

[cit15] Lishchuk A., Vasilev C., Johnson M. P., Hunter C. N., Törmä P., Leggett G. J. (2019). Turning the Challenge of Quantum Biology on Its Head: Biological Control of Quantum Optical Systems. Faraday Discuss..

[cit16] Besford Q. A., Yong H., Merlitz H., Christofferson A. J., Sommer J., Uhlmann P., Fery A. (2021). FRET-Integrated Polymer Brushes for Spatially Resolved Sensing of Changes in Polymer Conformation. Angew. Chem., Int. Ed..

[cit17] Besford Q. A., Merlitz H., Schubotz S., Yong H., Chae S., Schnepf M. J., Weiss A. C., Auernhammer G. K., Sommer J.-U., Uhlmann P., Fery A. (2022). Mechanofluorescent Polymer Brush Surfaces That Spatially Resolve Surface Solvation. ACS Nano.

[cit18] Tas S., Kopec M., van der Pol R., Cirelli M., de Vries I., Bölükbas D. A., Tempelman K., Benes N. E., Hempenius M. A., Vancso G. J., de Beer S. (2019). Chain End-Functionalized Polymer Brushes with Switchable Fluorescence Response. Macromol. Chem. Phys..

[cit19] Madsen J., Ducker R. E., Al Jaf O., Cartron M. L., Alswieleh A. M., Smith C. H., Hunter C. N., Armes S. P., Leggett G. J. (2018). Fabrication of Microstructured Binary Polymer Brush “Corrals” with Integral PH Sensing for Studies of Proton Transport in Model Membrane Systems. Chem. Sci..

[cit20] Poisson J., Hudson Z. M. (2022). Luminescent Surface-Tethered Polymer Brush Materials. Chem.–Eur. J..

[cit21] Arnold R. M., Patton D. L., Popik V. V., Locklin J. A. (2014). Dynamic Duo: Pairing Click Chemistry and Postpolymerization Modification To Design Complex Surfaces. Acc. Chem. Res..

[cit22] Neri-Cruz C. E., Teixeira F. M. E., Gautrot J. E. (2023). Guide to Functionalisation and Bioconjugation Strategies to Surface-Initiated Polymer Brushes. Chem. Commun..

[cit23] Jiang H., Xu F.-J. (2013). Biomolecule-Functionalized Polymer Brushes. Chem. Soc. Rev..

[cit24] Brotherton E. E., Johnson E. C., Smallridge M. J., Hammond D. B., Leggett G. J., Armes S. P. (2023). Hydrophilic Aldehyde-Functional Polymer Brushes: Synthesis, Characterization, and Potential Bioapplications. Macromolecules.

[cit25] Brotherton E. E., Jesson C. P., Warren N. J., Smallridge M. J., Armes S. P. (2021). New Aldehyde-Functional Methacrylic Water-Soluble Polymers. Angew. Chem., Int. Ed..

[cit26] Brotherton E. E., Smallridge M. J., Armes S. P. (2021). Aldehyde-Functional Diblock Copolymer Nano-Objects via RAFT Aqueous Dispersion Polymerization. Biomacromolecules.

[cit27] Brotherton E. E., Neal T. J., Kaldybekov S. B., Smallridge M. J., Khutoryanskiy V. V., Armes S. P. (2022). Aldehyde-Functional Thermoresponsive Diblock Copolymer Worm Gels Exhibit Strong Mucoadhesion. Chem. Sci..

[cit28] Brotherton E. E., Josland D., György C., Johnson E. C., Chan D. H. H., Smallridge M. J., Armes S. P. (2023). Histidine-Functionalized Diblock Copolymer Nanoparticles Exhibit Enhanced Adsorption onto Planar Stainless Steel. Macromol. Rapid Commun..

[cit29] Buksa H., Johnson E. C., Chan D. H. H., McBride R. J., Sanderson G., Corrigan R. M., Armes S. P. (2024). Arginine-Functional Methacrylic Block Copolymer Nanoparticles: Synthesis, Characterization, and Adsorption onto a Model Planar Substrate. Biomacromolecules.

[cit30] Johnson E. C., Varlas S., Norvilaite O., Neal T. J., Brotherton E. E., Sanderson G., Leggett G. J., Armes S. P. (2023). Adsorption of Aldehyde-Functional Diblock Copolymer Spheres onto Surface-Grafted Polymer Brushes via Dynamic Covalent Chemistry Enables Friction Modification. Chem. Mater..

[cit31] Wu T., Efimenko K., Genzer J. (2002). Combinatorial Study of the Mushroom-to-Brush Crossover in Surface Anchored Polyacrylamide. J. Am. Chem. Soc..

[cit32] Matyjaszewski K., Dong H., Jakubowski W., Pietrasik J., Kusumo A. (2007). Grafting from Surfaces for “Everyone”: ARGET ATRP in the Presence of Air. Langmuir.

[cit33] Robertson H., Gresham J. J., Nelson A. R. J., Prescott S. W., Webber G. B., Wanless E. J. (2024). Illuminating the Nanostructure of Diffuse Interfaces: Recent Advances and Future Directions in Reflectometry Techniques. Adv. Colloid Interface Sci..

[cit34] Bao Z., Bruening M. L., Baker G. L. (2006). Control of the Density of Polymer Brushes Prepared by Surface-Initiated Atom Transfer Radical Polymerization. Macromolecules.

[cit35] Clayden J. , Greeves N. and Warren S., Organic Chemistry, Oxford University Press, 2nd edn, 2012

[cit36] Filler M. A., Keung A. J., Porter D. W., Bent S. F. (2006). Formation of Surface-Bound Acyl Groups by Reaction of Acyl Halides on Ge(100)-2×1. J. Phys. Chem. B.

[cit37] Willott J. D., Murdoch T. J., Webber G. B., Wanless E. J. (2016). Nature of the Specific Anion Response of a Hydrophobic Weak Polyelectrolyte Brush Revealed by AFM Force Measurements. Macromolecules.

[cit38] Murdoch T. J., Humphreys B. A., Willott J. D., Prescott S. W., Nelson A., Webber G. B., Wanless E. J. (2017). Enhanced Specific Ion Effects in Ethylene Glycol-Based Thermoresponsive Polymer Brushes. J. Colloid Interface Sci..

[cit39] Murdoch T. J., Humphreys B. A., Johnson E. C., Prescott S. W., Nelson A., Wanless E. J., Webber G. B. (2018). The Role of Copolymer Composition on the Specific Ion and Thermo-Response of Ethylene Glycol-Based Brushes. Polymer.

[cit40] Brittain W. J., Minko S. (2007). Structural Definition of Polymer Brushes. J. Polym. Sci., Part A:Polym. Chem..

[cit41] Martinez A. P., Carrillo J. M. Y., Dobrynin A. V., Adamson D. H. (2016). Distribution of Chains in Polymer Brushes Produced by a “Grafting From” Mechanism. Macromolecules.

[cit42] Genzer J. (2006). In Silico Polymerization: Computer Simulation of Controlled Radical Polymerization in Bulk and on Flat Surfaces. Macromolecules.

[cit43] Zoppe J. O., Ataman N. C., Mocny P., Wang J., Moraes J., Klok H. A. (2017). Surface-Initiated Controlled Radical Polymerization: State-of-the-Art, Opportunities, and Challenges in Surface and Interface Engineering with Polymer Brushes. Chem. Rev..

[cit44] Pelter A., Rosser R. M., Mills S. (1984). Reductive Aminations of Ketones and Aldehydes Using Borane–Pyridine. J. Chem. Soc., Perkin Trans. 1.

[cit45] Abdel-Magid A. F., Carson K. G., Harris B. D., Maryanoff C. A., Shah R. D. (1996). Reductive Amination of Aldehydes and Ketones with Sodium Triacetoxyborohydride. Studies on Direct and Indirect Reductive Amination Procedures. J. Org. Chem..

[cit46] Mattson R. J., Pham K. M., Leuck D. J., Cowen K. A. (1990). An Improved Method for Reductive Alkylation of Amines Using Titanium(IV) Isopropoxide and Sodium Cyanoborohydride. J. Org. Chem..

[cit47] Gribble G. W., Lord P. D., Skotnicki J., Dietz S. E., Eaton J. T., Johnson J. (1974). Reactions of Sodium Borohydride in Acidic Media. I. Reduction of Indoles and Alkylation of Aromatic Amines with Carboxylic Acids. J. Am. Chem. Soc..

[cit48] Gajo C., Shchepanovska D., Jones J. F., Karras G., Malakar P., Greetham G. M., Hawkins O. A., Jordan C. J., Curchod B. F. E., Oliver T. A. A. (2024). Nile Red Fluorescence: Where's the Twist?. J. Phys. Chem. B.

[cit49] Golini C. M., Williams B. W., Foresman J. B. (1998). Further Solvatochromic, Thermochromic, and Theoretical Studies on Nile Red. J. Fluoresc..

[cit50] Ray A., Das S., Chattopadhyay N. (2019). Aggregation of Nile Red in Water: Prevention through Encapsulation in β-Cyclodextrin. ACS Omega.

[cit51] Pei L., Zhu H., Gong S., Dong W., Zhu L., Wang J. (2024). Comprehensive Analysis of Dye Adsorption and Supramolecular Interaction between Dyes and Cotton Fibers in Non-Aqueous Media/Less Water Dyeing System. Ind. Crops Prod..

[cit52] Lavis L. D., Rutkoski T. J., Raines R. T. (2007). Tuning the p *K*_a_ of Fluorescein to Optimize Binding Assays. Anal. Chem..

[cit53] Hwang J. Y., Shim S., Hwang G. T. (2018). 4′,5′-Bis(Dimethylamino)Fluorescein Exhibits PH-Dependent Emission Behavior Distinct From That of Fluorescein. Asian J. Org. Chem..

[cit54] Shen Q., Fang C., Hu L., Serpe M. (2024). Fluorescent Nile Blue-functionalized Poly (*N* -isopropylacrylamide) Microgels Responsive to Temperature and Polyamines. SmartMat.

[cit55] Li Y., Bai X., Yang D. (2024). Development and Application of Cationic Nile Blue Probes in Live-Cell Super-Resolution Imaging and Specific Targeting to Mitochondria. ACS Cent. Sci..

[cit56] Zhao Y., Truhlar D. G. (2008). The M06 Suite of Density Functionals for Main Group Thermochemistry, Thermochemical Kinetics, Noncovalent Interactions, Excited States, and Transition Elements: Two New Functionals and Systematic Testing of Four M06-Class Functionals and 12 Other Functionals. Theor. Chem. Acc..

[cit57] Ma H., He J., Liu X., Gan J., Jin G., Zhou J. (2010). Surface Initiated Polymerization from Substrates of Low Initiator Density and Its Applications in Biosensors. ACS Appl. Mater. Interfaces.

[cit58] Jonas A. M., Glinel K., Oren R., Nysten B., Huck W. T. S. (2007). Thermo-Responsive Polymer Brushes with Tunable Collapse Temperatures in the Physiological Range. Macromolecules.

[cit59] Xue C., Yonet-Tanyeri N., Brouette N., Sferrazza M. V., Braun P. E., Leckband D. E. (2011). Protein Adsorption on Poly(N-Isopropylacrylamide) Brushes: Dependence on Grafting Density and Chain Collapse. Langmuir.

[cit60] Plunkett K. N., Zhu X., Moore J. S., Leckband D. E. (2006). PNIPAM Chain Collapse Depends on the Molecular Weight and Grafting Density. Langmuir.

[cit61] Teunissen L. W., Kuzmyn A. R., Ruggeri F. S., Smulders M. M., Zuilhof H. (2022). Thermoresponsive, Pyrrolidone-Based Antifouling Polymer Brushes. Adv. Mater. Interfaces.

[cit62] Reichardt C. (1965). Empirical Parameters of the Polarity of Solvents. Angew. Chem., Int. Ed..

[cit63] Cheng N., Bao P., Evans S. D., Leggett G. J., Armes S. P. (2015). Facile Formation of Highly Mobile Supported Lipid Bilayers on Surface-Quaternized PH-Responsive Polymer Brushes. Macromolecules.

[cit64] Greenspan P., Fowler S. D. (1985). Spectrofluorometric Studies of the Lipid Probe, Nile Red. J. Lipid Res..

[cit65] Kim H.-H., Song N. W., Park T. S., Yoon M. (2006). Laser Scanning Confocal Microscope (LSCM)-Fluorescence Spectral Properties of Nile Red Embedded in Polystyrene Film of Different Thickness. Chem. Phys. Lett..

[cit66] Kucherak O. A., Oncul S., Darwich Z., Yushchenko D. A., Arntz Y., Didier P., Mély Y., Klymchenko A. S. (2010). Switchable Nile Red-Based Probe for Cholesterol and Lipid Order at the Outer Leaflet of Biomembranes. J. Am. Chem. Soc..

[cit67] Dutta A. K., Kamada K., Ohta K. (1996). Spectroscopic Studies of Nile Red in Organic Solvents and Polymers. J. Photochem. Photobiol., A.

[cit68] Torrado B., Pannunzio B., Malacrida L., Digman M. A. (2024). Fluorescence Lifetime Imaging Microscopy. Nat. Rev. Methods Primers.

[cit69] Krishna M. M. G. (1999). Excited-State Kinetics of the Hydrophobic Probe Nile Red in Membranes and Micelles. J. Phys. Chem. A.

[cit70] Norman K., Ghanbari-Siahkali A., Larsen N. B. (2005). 6 Studies of Spin-Coated Polymer Films. Annu. Rep. Prog. Chem., Sect. C:Phys. Chem..

