# Correction: Rational design of pigment–polymer antenna complexes

**DOI:** 10.1039/d6sc90162j

**Published:** 2026-08-24

**Authors:** Edwin C. Johnson, Demetris Bates, Tingxiang Yang, Kasimir Gregory, Jodie West, Deborah B. Hammond, James P. Pidgeon, Jenny Clark, Nicholas H. Williams, C. Neil Hunter, Steven P. Armes, Graham J. Leggett

**Affiliations:** a School of Mathematical and Physical Sciences, University of Sheffield Dainton Building, Brook Hill Sheffield S3 7HF UK Graham.Leggett@sheffield.ac.uk; b School of Biosciences, University of Sheffield Firth Court Sheffield S10 2TN UK; c School of Science and Technology, University of New England Armidale NSW 2351 Australia

## Abstract

Correction for ‘Rational design of pigment–polymer antenna complexes’ by Edwin C. Johnson *et al.*, *Chem. Sci.*, 2026, **17**, 11566–11579, https://doi.org/10.1039/d6sc01202g.

An incorrect URL for the University of Sheffield's Online Data Archive (ORDA) was given in the originally published manuscript. The updated data availability statement is given as follows:

**Data availability**

Data for this paper are available at the University of Sheffield's Online Data Archive (ORDA) at https://doi.org/10.15131/shef.data.31230256.

Supplementary information (SI): materials and characterization techniques, synthetic protocols, characterization data for NBet, high resolution XPS spectra recorded for BiBB/BB functionalized silicon wafers and PAGEO5MA brushes conjugated to a range of amine-functional dyes. Absorbance spectra recorded for 6-aminoerythrosine, 6-aminofluorescein and NBet in solution. Unnormalized absorbance spectra recorded for P(NRet)AGEO5MA brushes and emission data for the same films recorded over an extended range of concentrations. See DOI: https://doi.org/10.1039/d6sc01202g.

The Royal Society of Chemistry apologises for these errors and any consequent inconvenience to authors and readers.

