# Supplementary material for: Rational design of pigment–polymer antenna complexes
Source: Chem Sci. 2026 Apr 24;17(23):11566–79. doi: 10.1039/d6sc01202g (PMC13140099; doi:10.1039/d6sc01202g)
Supplement: SC-017-D6SC01202G-s001 [file SC-017-D6SC01202G-s001.pdf]

Supplementary Information (SI) for Chemical Science.
This journal is © The Royal Society of Chemistry 2026

Supporting Information for:

Rational design of pigment-polymer antenna complexes

Edwin C. Johnson[a†], Demetris Bates[a], Tingxiang Yang[b], Kasimir Gregory[c], Jodie West, Deborah B. Hammond[a], James P. Pidgeon[a], Jenny Clark[a], Nicholas H. Williams,[a] C. Neil Hunter[b], Steven P. Armes[a], Graham Leggett[a]*.

[a] School of Mathematical and Physical Sciences, University of Sheffield, Dainton Building, Brook Hill, Sheffield, S3 7HF, UK

[b] School of Biosciences, University of Sheffield, Firth Court, Sheffield, S10 2TN, UK

[c] School of Science and Technology, University of New England, Armidale, NSW 2351, Australia

[†]Present address: College of Science, Engineering and Environment, University of Newcastle, Callaghan, NSW 2308, Australia

Contents

Experimental Section 2

Materials 2

Synthetic Protocols 3

Characterization Techniques 8

Computational techniques 10

Supplementary characterization data for the Nile Blue ethylamine synthesis**Error! Bookmark not defined.**

Absorbance spectra of dye-functionalized brushes and non-conjugated dyes 11

XPS spectra of dye-functionalized brushes 13

Nile Red concentration in brush as a function of degree of functionalization 17

Absorbance spectra of Nile Red-functionalized PAGEO5MA brushes 18

Fluorescence spectra of Nile Red-functionalized PAGEO5MA brushes 19

References 20

Experimental Section

Materials

Sodium periodate (≥99.8%, $NaIO_4$), (3-aminopropyl)triethoxysilane (>99%, APTES), triethylamine (99%, NEt_3), 2-bromoisobutyryl bromide (>99%, BIBB), copper(II) chloride (99.999%, $CuCl_2$), ascorbic acid (>98%, AscA), sodium cyanoborohydride (95%, $NaBH_3CN$), dichloromethane (>99%, DCM), diethyl ether (≥99.8%), *N*-(1-naphthyl)ethylenediamindihydrochloride (>98%), benzoyl bromide (BB, 97%), 6-aminofluorescein (isomer I), 6-aminoerythrosin, 1-naphthylamine (97%), 1-aminopyrene (97%), 1-pyrenemethylamine hydrochloride (95%) and ammonia solution (NH_4OH, 35%) were purchased from Sigma-Aldrich, UK and were used without further purification. 3-Diethylaminophenol (99%, Acros Organics, UK), sodium azide (>99%, Acros Organics, UK), sodium nitrite (>97%, Alfa Aesar, UK), dibromoethane (98%, Alfa Aesar, UK), hydrochloric acid (HCl, 35 wt%, Scientific Laboratory Supplies, UK), potassium carbonate (100%, K_2CO_3, VWR, UK), petroleum ether (40-60, 95% VWR), magnesium sulfate (dried, Fisher Scientific, UK), sodium hydroxide (>97%, NaOH, Fisher Scientific, UK), sodium chloride (>=99.5%, NaCl, Fisher Scientific, UK), 1-methylnaphthalene (96%, Fisher Scientific, UK) were also used as purchased. GEO5MA monomer was kindly donated by GEO Specialty Chemicals, UK and was used without further purification. All other solvents were purchased from Fisher Scientific, UK and were used as received unless otherwise stated herein. *N,N,N′,N″,N″*-pentamethyldiethylenetriamine (>99%, PMDETA) was also purchased from Fisher Scientific, UK.

Deionized water (pH 6.8) obtained from an Elga Elgastat Oprion 3A water purification system was used in all experiments unless otherwise stated. Native oxide-coated silicon wafers were purchased from Pi-KEM (Peterborough, UK). Copper/palladium grids were purchased from Agar Scientific, UK. Column chromatography was performed on silica gel (40-60 μm, VWR, UK), monitored by thin layer chromatography (TLC) using Merck silica gel 60 F_{254} plates and visualized under UV light.

^1H NMR spectra were recorded on a Bruker Avance III HD and chemical shifts referenced to residual solvent. Mass spectra were recorded at the University of Sheffield Mass Spectrometry facility using either an Agilent 6530 Accurate Mass LC-MS QToF mass spectrometer (LCMS, high resolution) or a Waters LCT Classic (direct infusion, low resolution) instrument. 3-Diethylamino-2-nitrosophenol hydrochloride[1] and 2-hydroxy-

functionalized Nile Red[2] were synthesized according to published protocols. Microscope coverslip glass slides (22 mm, 50 mm, 1.5 mm thickness) were obtained from Menzel-Gläser, Germany. Gold wire (99.997% trace metals basis, Goodfellow Advanced Materials, UK) and chromium chips (99.5% trace metals basis, Sigma-Aldrich, UK) were used for thermal evaporation.

Synthetic Protocols

Preparation of 2-aminoethyl-Nile Blue (NBet)

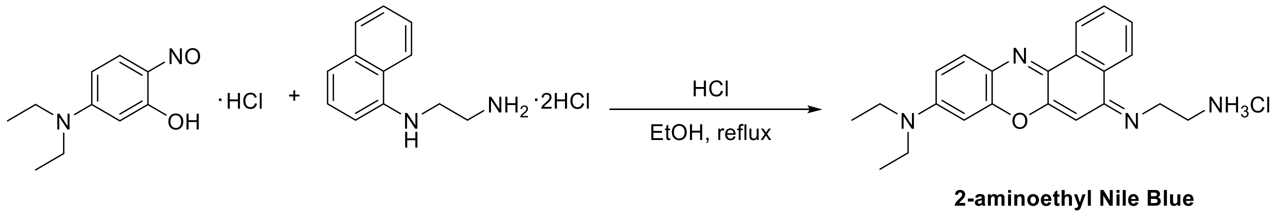

Scheme 1. Synthesis of 2-aminoethyl-functionalized Nile Blue dye.

5-Diethylamino-2-nitrosophenol hydrochloride (0.445 g, 1.93 mmol) and *N*-(1-naphthyl)ethylenediamine dihydrochloride (0.500 g, 1.93 mmol) were suspended in ethanol (10 mL). Then HCl (34 µL, 11.3 M, 0.38 mmol) was added and the reaction mixture heated to reflux for 1.5 h. After cooling to 20°C, the resulting precipitate was isolated by vacuum filtration and dissolved in methanol; the crude product was purified by column chromatography (3:1 chloroform/methanol eluent) yielding a dark blue solid (0.593 g, 77%). This product was dissolved in methanol, acidified using 1 M HCl and dried prior to analysis: ^{1}H NMR (CD_3OD, 400 MHz): δ = 8.87 (dd, J = 8.1, 0.9 Hz, 1H), 8.49 (d, 8.2 Hz, 1H), 7.95-7.90 (m, 1H), 7.88 (d, 9.5 Hz, 1H), 7.86-7.81 (m, 1H), 7.39 (dd, J = 9.5, 2.7 Hz, 1H), 7.11 (s, 1H), 6.98 (d, 2.6 Hz, 1H), 4.10 (t, J = 6.4 Hz, 2H), 3.77 (q, 7.2 Hz, 4H), 3.47 (t, 6.3 Hz, 2H), 1.39 (t, 7.1 Hz, 6H) ppm; $^{13}C\{^{1}H\}$ NMR (CD_3OD, 100 MHz): δ = 157.2, 154.9, 151.1, 148.3, 133.0, 132.1, 131.5, 131.4, 130.7, 129.2, 123.7, 123.1, 122.8, 116.6, 95.6, 93.0, 46.0, 41.3, 37.7, 11.8 ppm; HRMS (ESI): *m/z* 361.2028 $[M]^+$, calculated for $C_{22}H_{25}N_4O^+$ 361.2023.

Synthesis of 2-(2-aminoethoxy)Nile Red (NRet)

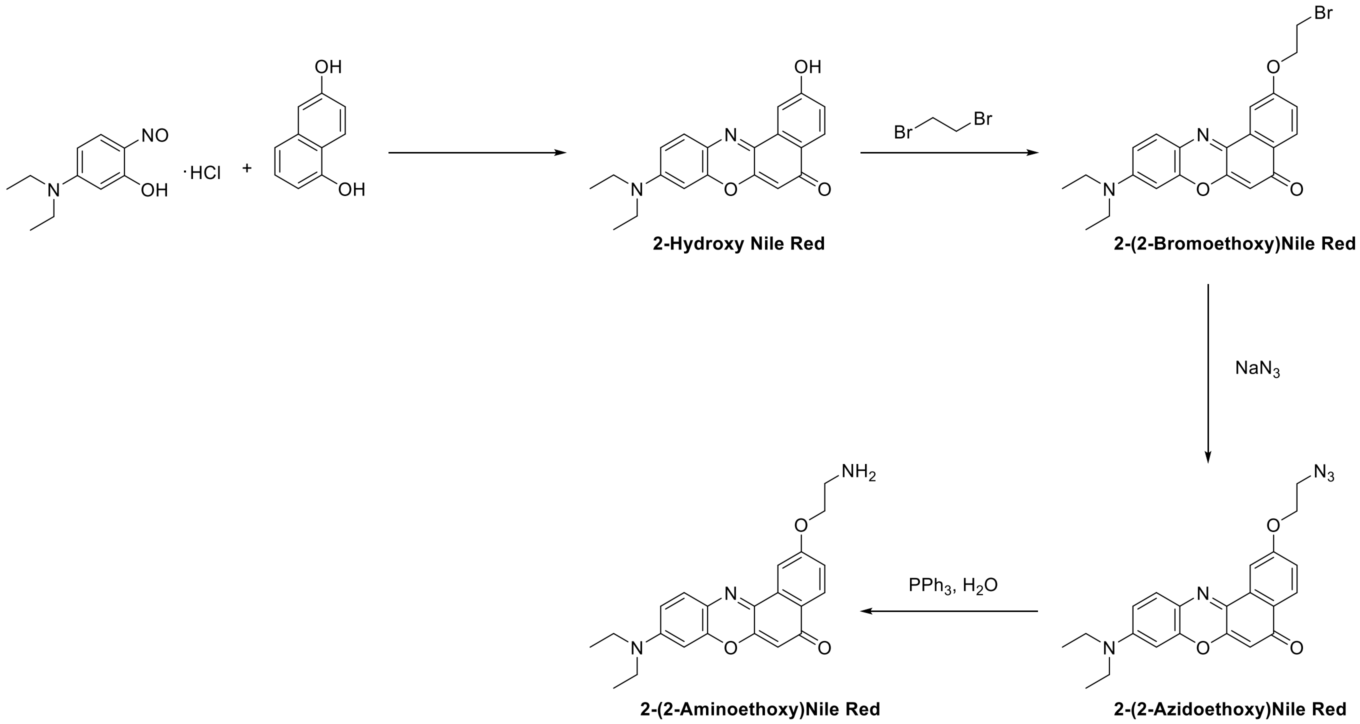

Scheme 2. Synthesis of 2-(2-Aminoethoxy)Nile Red

Preparation of 2-(2-bromoethoxy)Nile Red

The synthesis described below was adapted from a literature protocol.[3] (2-HydroxyNile Red (0.407 g, 1.22 mmol), potassium carbonate (1.686 g, 12.20 mmol) and anhydrous DMF (10 mL) were combined under a N_2 atmosphere. 1,2-Dibromoethane (3.2 mL, 37.13 mmol) was added to the stirred mixture and the reaction mixture was heated to 65 °C for 70 min. Reaction progress was monitored by TLC analysis (1:1 ethyl acetate/petroleum ether) and the flask was cooled to 20°C. Dichloromethane (50 mL) and water (50 mL) were added and the organic layer was washed with water (2 x 30 mL). These aqueous solutions were combined and extracted with dichloromethane (3 x 30 mL), then the combined organic layers were washed with saturated aqueous brine (80 mL), dried over $MgSO_4$ and concentrated *in vacuo*. The crude solid was subjected to flash chromatography (initially 1:1 ethyl acetate/petroleum ether, increasing to 2:1 once impurities had eluted) yielding 2-(2-bromoethoxy)-Nile Red as a purple solid (0.292 g, 0.66 mmol, 54%): ^{1}H NMR (400 MHz, $CDCl_3$) δ 8.27 (d, J = 8.7 Hz, 1H), 8.10 (d, J = 2.6 Hz, 1H), 7.64 (d, J = 9.1 Hz, 1H), 7.23 (dd, J = 8.7, 2.6 Hz, 1H), 6.70 (dd, J = 9.1, 2.8 Hz, 1H), 6.50 (d, J = 2.7 Hz, 1H), 6.34 (s, 1H), 4.54 (t, J = 6.2 Hz, 2H), 3.76 (t, J = 6.2 Hz, 2H), 3.50 (q, J = 7.1 Hz, 4H), 1.29 (t, J = 7.1 Hz, 6H); ^{13}C{^{1}H} NMR (100 MHz, $CDCl_3$) δ 183.09, 160.67, 152.09, 150.82, 146.87, 139.67, 134.08, 131.10, 127.91, 126.17, 124.71,

118.32, 109.61, 106.65, 105.28, 96.28, 77.37, 77.26, 77.05, 76.73, 68.06, 45.11, 28.97, 12.65; HRMS (ESI): *m/z* 441.0818 [M+H]$^+$, calculated for $C_{22}H_{22}N_2O_3Br^+$ 441.0808.

Preparation of 2-(2-azidoethoxy)-Nile Red

The synthesis described below was adapted from a literature protocol.[4] 2-(2-Bromoethoxy)-Nile Red (0.290 g, 0.657 mmol) and NaN_3 (0.064 g, 0.984 mmol) were co-dissolved in anhydrous DMF (10 mL). This reaction mixture was stirred at 80 °C under N_2 for 20 h and cooled to 20 °C. Ethyl acetate (50 mL) was added and the solution was washed with 3 M NaOH (3 x 50 mL) and brine (50 mL). The organic layers were combined, dried over $MgSO_4$ and concentrated *in vacuo*, yielding 2-(2-azidoethoxy)-Nile Red as a purple solid (0.261 g, 0.647 mmol, 98%). This precursor was used without further purification. ^{1}H NMR (400 MHz, $CDCl_3$) δ = 8.24 (d, *J* = 8.7 Hz, 1H), 8.05 (d, *J* = 2.7 Hz, 1H), 7.58 (d, *J* = 9.0 Hz, 1H), 7.20 (dd, *J* = 8.7, 2.6 Hz, 1H), 6.66 (dd, *J* = 9.1, 2.7 Hz, 1H), 6.45 (d, *J* = 2.7 Hz, 1H), 6.30 (s, 1H), 4.38 (t, *J* = 4.9 Hz, 2H), 3.71 (t, *J* = 5.0 Hz, 2H), 3.48 (q, *J* = 7.1 Hz, 4H), 1.28 (t, *J* = 7.1 Hz, 6H) ppm; ^{13}C{^{1}H} NMR (100 MHz, $CDCl_3$) δ = 183.11, 160.80, 152.09, 150.82, 146.87, 139.69, 134.08, 131.09, 127.89, 126.16, 124.71, 118.38, 109.60, 106.44, 105.29, 96.28, 67.24, 50.18, 45.10, 12.64 ppm; HRMS (ESI): *m/z* 404.1722 [M+H]$^+$, calcd for $C_{22}H_{22}N_5O_3^+$ 404.1717.

Preparation of 2-(2-aminoethoxy)-Nile Red (NRet)

The synthesis described below was adapted from a literature protocol.[5] 2-(2-Azidoethoxy)-Nile Red (0.030 g, 0.074 mmol) and PPh_3 (0.029 g, 0.111 mmol) were co-dissolved in anhydrous THF (1 mL) and stirred under N_2 for 1 h at 20 °C. Water (5 drops) was added, the reaction mixture was stirred for 18 h at 20 °C and the solvent was removed *in vacuo*. The crude material was subjected to flash chromatography (initially 95:5 dichloromethane/methanol/1% NH_4OH, rising to 10% methanol once impurities had eluted) yielding 2-(2-aminoethoxy)-Nile Red as a purple solid (0.022 g, 0.058 mmol, 78%): ^{1}H NMR (400 MHz, $CDCl_3$) δ 8.20 (d, J = 8.7 Hz, 1H), 8.03 (d, J = 2.6 Hz, 1H), 7.55 (d, J = 9.1 Hz, 1H), 7.16 (dd, J = 8.7, 2.6 Hz, 1H), 6.62 (dd, J = 9.1, 2.7 Hz, 1H), 6.41 (d, J = 2.7 Hz, 1H), 6.27 (s, 1H), 4.20 (t, J = 5.2 Hz, 2H), 3.45 (q, J = 7.1 Hz, 4H), 3.18 (t, J = 5.2 Hz, 2H), 1.26 (t, J = 7.1 Hz, 7H), MS (ESI): *m/z* 378.2 [M+H]$^+$, calcd. for $C_{22}H_{24}N_3O_3^+$ 378.2.

Film Formation

Surface functionalization of glass and silicon substrates with ATRP initiator

Functionalization of planar silicon wafers with ATRP initiator was achieved using a modified experimental protocol reported by Johnson *et al*.[6] Silicon (100) wafers were cut into small pieces (~1 × 1 cm^2) before being exposed to ozone during UV irradiation for 30 min using a Bioforce Nanosciences ProCleaner. The resulting wafers were then placed in an open petri dish along with a 3 mL glass vial containing ~100 µL APTES. The petri dish was then placed in a desiccator, which was subsequently sealed and placed under vacuum. Vapor deposition was allowed to proceed for 30 min at 20 °C before the wafers were removed and placed in a 110 °C oven for 30 min. The wafers were then functionalized by immersion in dichloromethane followed by sequential addition of NEt_3 (final concentration = 0.2 mM) and BiBB (final concentration = 0.2 mM), and allowing amidation of the surface primary amine groups to proceed for 1 h at 22 °C. Finally, the ATRP initiator-functionalized silicon wafers were rinsed extensively with ethanol and water, before drying under a stream of compressed air.

Control of initiator density on *glass and silicon* surfaces

Controlling the surface density of initiator groups enables brushes to be prepared over a range of grafting densities. For silicon wafer and glass substrates, this involved modifying the above protocol to include a diluent, benzoyl bromide (BB), during amidation. Substrates were immersed in DCM before the addition of NEt_3 and a premixed solution of initiator (BiBB) and diluent (BB). The overall concentration of initiator and diluent was kept constant (0.2 M) while the molar ratio of these two reagents was varied.

Grafting of PGEO5MA brushes *via* surface-initiated ARGET ATRP from initiator functionalised surfaces

SI-ARGET ATRP was employed to grow PGEO5MA brushes from each planar substrate. We have previously reported a detailed synthetic protocol.[7] Briefly, a GEO5MA: $CuCl_2$: PMDETA: ascorbic acid molar ratio of 1000: 1: 5: 10 and a 45% v/v aqueous monomer solution was utilized. Polymerizations were allowed to proceed for 1 h at 22 °C in all cases. Each PGEO5MA-functionalized wafer was subsequently rinsed thoroughly with ethanol and water and then dried using a stream of N_2 gas.

Selective oxidation of *cis*-diol-functional PGEO5MA brushes for the synthesis of aldehyde-functional PAGEO5MA brushes

Our previously reported experimental protocol for selective oxidation of surface-grafted polymer brushes was employed.[7] PGEO5MA-functionalized planar silicon wafers were immersed in a 3.0 g dm^{-3} aqueous solution of $NaIO_4$ for 30 min at 22 °C, targeting a degree of oxidation of 100% in all cases. Each PAGEO5MA-functionalized silicon wafer was subsequently rinsed thoroughly with water and then dried using a stream of compressed air.

Preparation of dye-functionalized PAGEO5MA brushes from aldehyde-functional PAGEO5MA brushes *via* reductive amination

PAGEO5MA brushes were functionalized with the reactive amine dyes *via* reductive amination as shown in Scheme 1, main MS. A 5 μM solution (volume = 5 mL) of the reactive dye was prepared in water, methanol, ethanol, or THF followed by addition of a 1.5 × molar excess of $NaBH_3CN$ which was added as a solid. Brush-functionalized silicon wafers were immersed into this solution and allowed to react for 24 h at 50 °C. The resulting dye-functionalized brushes were rinsed extensively using methanol, ethanol and water and then dried under a stream of compressed air.

Preparation of spin-cast films

Solid spun-cast samples were produced using soda-lime glass coverslips (22×22 mm, 0.13-0.17 mm thickness, MCS22222, Fisherbrand) as substrates. These were cleaned beforehand by sonicating for 10 minutes in ~1% Hellmanex III (9-307-011-4-507, Hellma) in deionised water (nominally 15 MΩ cm, DV 25 PURELAB Option, Elga), then sonicated in successive neat cleaning solvents – deionised water, acetone (≥99.5%, ACS reagent, 179124-2.5L, Sigma-Aldrich), and then isopropanol (≥99.5%, HPLC grade, P/7507/17, Fisher Scientific) – and then blow-dried with a nitrogen gun. The substrates were finally subject to UV-ozone treatment (UV.TC.220, BioForce Nanosciences) for ~30 min prior to spin-casting.

To produce dye-dispersed polymer matrices, poly(methyl methacrylate) (PMMA; average Mw ~350000, 445746-25G, Aldrich) was left to dissolve at room temperature in DMF overnight, yielding a 50 mg mL^{-1} stock solution. Different volumes of this stock solution and dye in DMF were mixed to make the dye-to-PMMA ratios required for the different concentrations of the final solid films. These mixed solutions were used to make the thin spun-cast films by using a spin-coater (WS-400BZ-6NPP/LITE or WS-650MZ-23NPP/LITE, Laurel). To prevent the adhering vacuum from bowing (distorting) the thin coverslip substrates, a relatively thick

microscope slide is attached to the vacuum chuck, with the coverslip taped on using double-sided tape (3M Company). The chuck, microscope slide, and the taped-on coverslip are spun together, and allowed to reach the desired spin speed before deposition of the solution. The mixed solutions were dynamically pipetted onto the spinning coverslips and left spinning for at least 30 s at 2000 rpm. Upon completion, the coverslips are taken off the microscope slide and residual tape removed using a razor and tweezers.

To determine the concentration of Nile Red in spin-cast films, the thickness was determined by using atomic force microscopy to measure the depth of a scratch formed in the polymer film. UV-vis spectra were acquired for spin-cast films and the concentration determined from the Beer-Lambert law, using the film thickness as the path-length.

Characterization Techniques

Ellipsometry

Measurements

Ellipsometry measurements on dry polymer brushes grown from planar silicon wafers were performed in air at 20 °C using a J. A. Woollam M-2000 V ellipsometer at a fixed angle of incidence of 75° normal to the sample surface. A wavelength range of λ = 370 to 1000 nm was used to obtain Ψ and Δ. The ellipsometer set-up enabled a relatively large sampling area of approximately 0.5 cm × 1 cm, which corresponds to around 50% of the total surface area of each brush-functionalized wafer

In situ ellipsometry measurements were conducted in a Woollam large volume, solid–liquid fluid cell with optical glass windows at 65° using the same J. A. Woollam M-2000 V ellipsometer. Cauchy parameters for water, methanol, ethanol and THF were determined by measuring Ψ and Δ for a reference bare silicon wafer with a known surface thickness of silica (1.5 nm). A PGEO5MA or PAGEO5MA brush-functionalized wafer (40 nm dry brush thickness) was installed in the cell before filling with each solvent in turn and determining the ellipsometric parameters over the same wavelength range.

Modeling

Data analysis and modeling were performed using Woollam CompleteEase software, which fits the Ψ and Δ values to the experimental data.

Dry PAGEO5MA brushes prior to dye functionalization were modelled with a two-layer model consisting of a native oxide layer and Cauchy layer with constants of A_n = 1.459, B_n = 0.006,

and $C_n = 0$. Solvated brushes were analyzed using a similar model. However, in this case, the fronting medium was water, whereas air was used for the dry measurements. The solvated brush layer was modeled using an effective medium approximation (EMA) where optical properties are calculated as a weighted average according to the relative volume fractions of the polymer (Cauchy layer as described above) plus solvent (assuming a Cauchy layer with the parameters determined above).

X-Ray Photoelectron Spectroscopy

X-ray photoelectron spectroscopic (XPS) analysis of dry polymer brushes grafted from planar silicon wafers was performed using a Kratos Axis Supra spectrometer. Step sizes of 0.50 and 0.10 eV were used to record survey and high-resolution C1s, O1s and N1s spectra, respectively. In each case, spectra were recorded from at least two separate areas for each surface-grafted brush. The XPS data were analyzed using Casa XPS software. All binding energies were calibrated with respect to the C1s saturated hydrocarbon peak at 285.0 eV.

For depth-profiling, an Ar_{2000}^+ giant gas cluster ion source was used with a beam energy of 10 keV and an ion current of 8 nA. The beam was rastered across an area 2 $\times$ 2 mm^2. XPS spectra are first collected from the analysis area before any etching has occurred. X-ray data are collected from an area 110 mm in diameter at the centre of the etch crater, with an X-ray emission current of 25 mA. The sample is then etched for a fixed period, before the beam is blanked and the XPS analysis is repeated. This sequence of etching followed by analysis is continued for a prescribed number of depths.

UV-Visible Absorption Spectroscopy

UV-visible absorption spectra were recorded at normal incidence for a series of dye solutions and the corresponding dye-conjugated brush-functionalized glass coverslips. Measurements were performed using a Cary 50 UV-visible spectrophotometer (Agilent Technologies, USA) . using a quartz cuvette cell (10 mm path length). Baselines were recorded using a solvent-filled (or empty for dry brush measurements) cuvette cell prior to measurements. Absorbance was measured from 300 to 800 nm. For measurements on the dye-conjugated brush-functionalized wafers, each wafer was placed in a cuvette and positioned in line of the beam. Prior to measurements, each brush-functionalized wafer was immersed in water and the solution pH was adjusted using either HCl or NaOH prior to drying under a stream of N_2 gas. A background subtraction was performed after each measurement using a bare glass coverslip.

Fluorescence Measurements

Fluorescence emission spectroscopy and Fluorescence Lifetime Microscopy (FLIM) Fluorescence emission spectra were obtained using an FLS3-22-320 spectrofluorimeter (Horiba Jobin Yvon). equipped with a 450 W xenon arc lamp. Samples were excited at 480 nm using 10 nm entrance and exit slit bandpass to ensure adequate excitation intensity. Emission signals were collected with a 5 nm bandpass slit over the wavelength range pf 505 – 800 nm Fluorescence lifetimes were recorded using a home-built time-resolved fluorescence microscope. A 485 nm picosecond diode laser (PicoQuant, PDL 828) served as the excitation source for lifetime measurements. The excitation beam was focused onto the sample surface with a 20x/0.5 HD air objective (ZEISS, EC Epiplan-NEOFLUAR). To suppress scattered excitation light, the signal was passed through a 550nm long-pass filter before being detected. For lifetime measurements, fluorescence photons were detected with a detector (HPM-100-50, Becker & Hickl) connected to a time-correlated single-photon counting (TCSPC) module (SPC-150, Becker & Hickl). The laser modulation was synchronized with the TCSPC system, which triggered the laser pulse and registered photon arrival times relative to the excitation source. The instrument response function (IRF) of the system is ~ 160 ps. The recorded data was processed with FLIMfit 5.1.1 and OriginPro 2024 software.

Computational techniques

WFT calculations of the molecular volume of each dye

The partial charge and atomic radii used to calculate radial charge densities, were calculated on isolated dye molecules using a density-derived electrostatic and chemical (DDEC6) charge distribution analysis.[8] Atomic $\langle r^3 \rangle$ moments from DDEC6 charge partitioning were used as an electron-density–based proxy for atomic size, reflecting the volumetric extent of each atom’s electron distribution within the molecule. The level of theory was M06-2X/def2-SVPD for the larger dye molecules.[9,10]

XPS spectra for initiator-functionalized surfaces

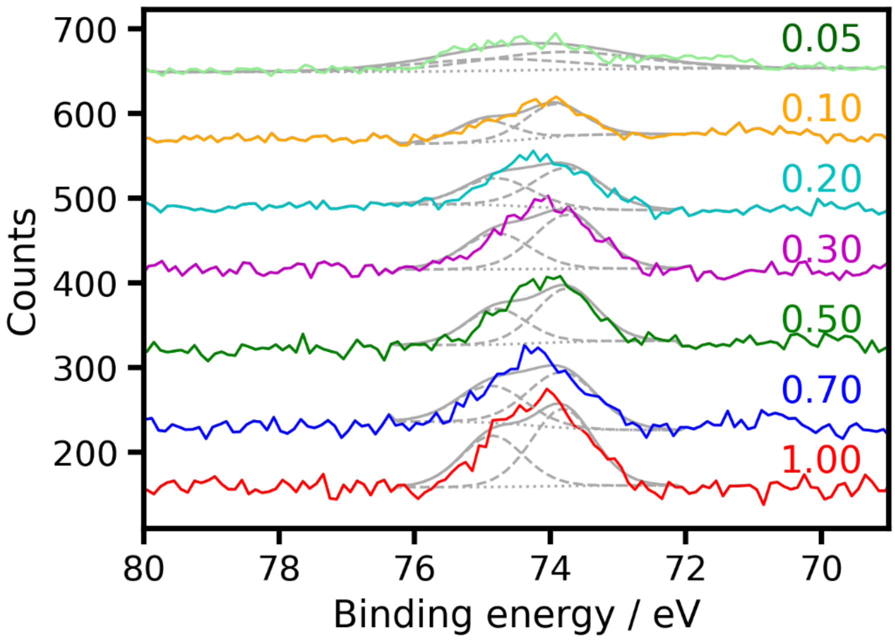

Figure S1. Br3d XPS spectra recorded for BiBB/BB functionalized silicon wafers. BiBB/BB molar ratio indicated for each spectrum. Data offset by 100 counts for clarity. Spectra were fitted with a doublet with a fixed height ratio of 0.66.

Absorption spectra recorded for dyes in solution

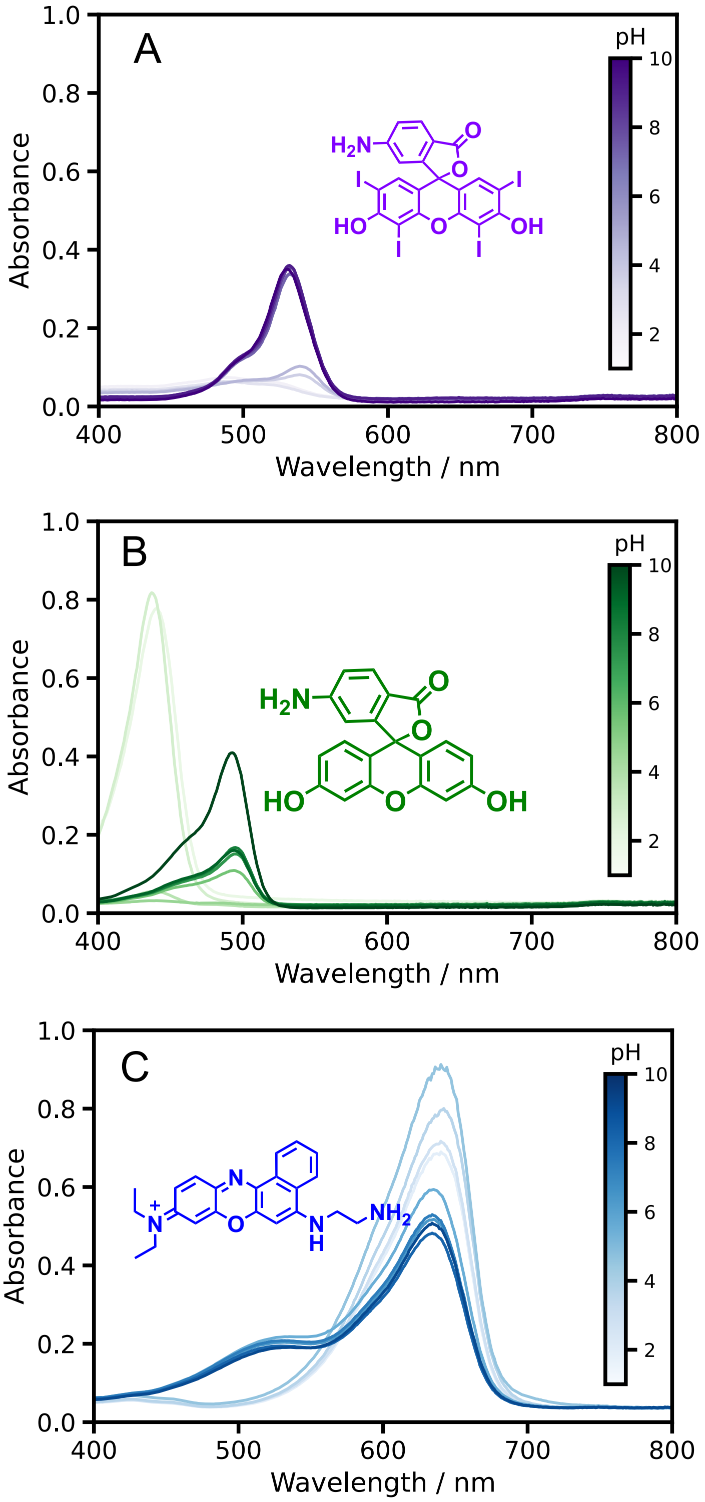

Figure S2. Absorption spectra recorded for various dyes dissolved in methanol. The pH was adjusted by adding HCl or NaOH.

XPS spectra recorded for dye-functionalized brushes

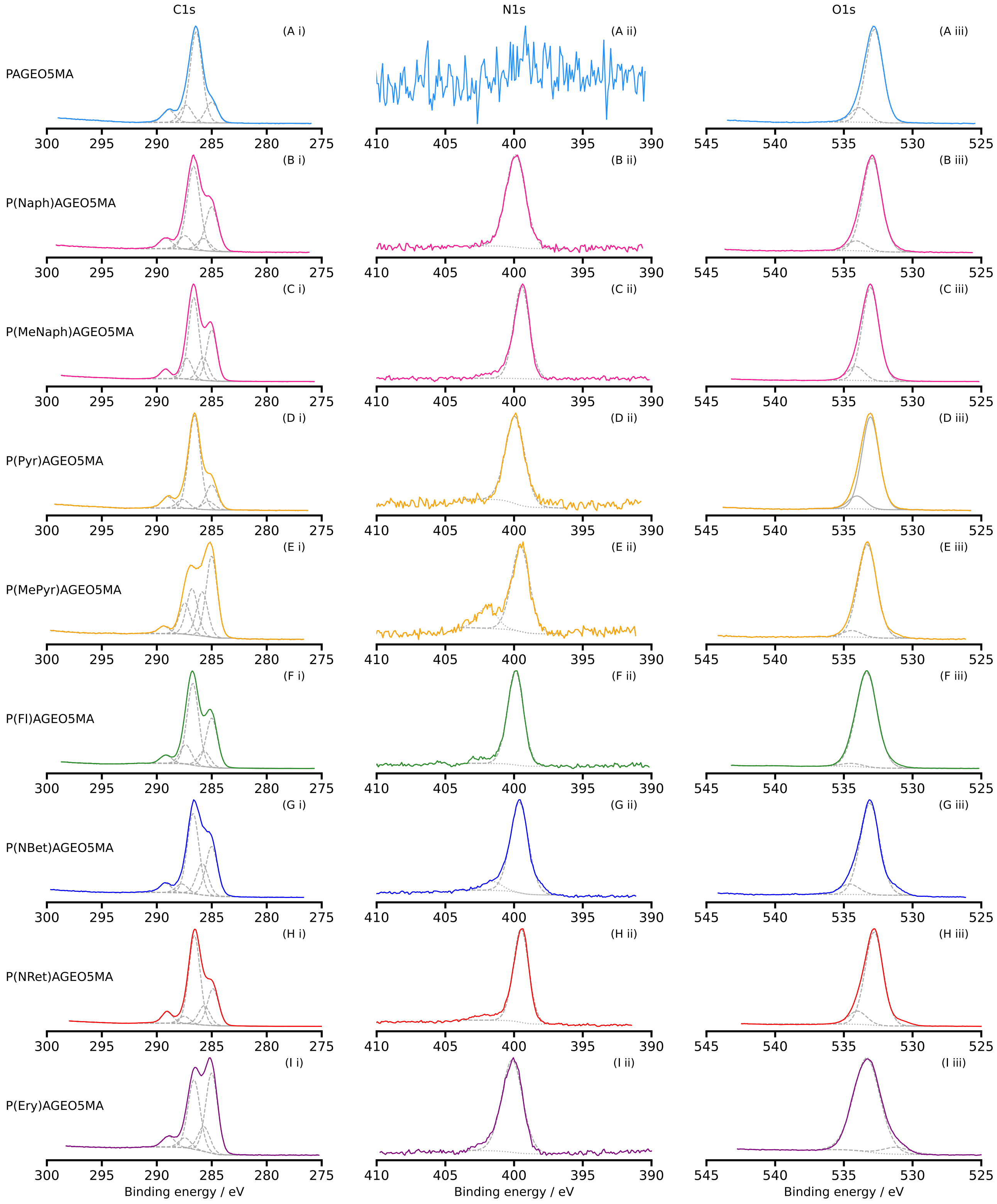

Figure S3. High resolution C1s (left), N1s (middle) and O1s XPS spectra recorded for PAGEO5MA brushes conjugated with eight different reactive dyes (see Scheme 1). Colored traces are the raw data, with fitted components shown in grey dashed lines.

Degree of functionalization of PAGEO5MA brushes with Nile Red

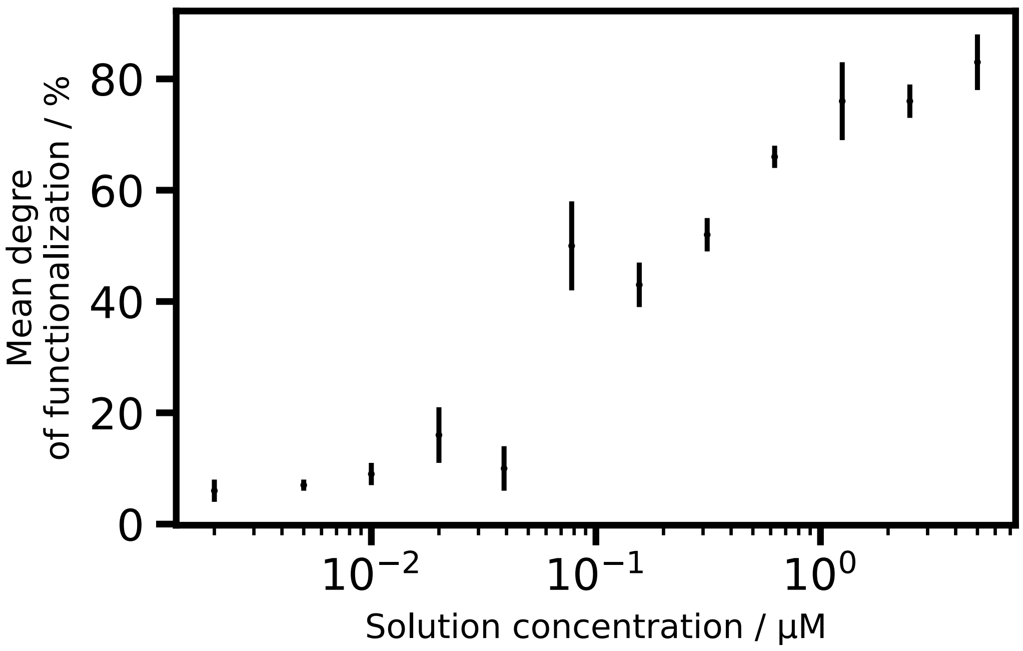

Figure S4. Variation in the mean degree of functionalization (black data points) and dye concentration within fully-dense PAGEO5MA brushes (red data points) with NRet solution concentration. The mean degree of brush functionalization was determined via XPS.

Variable Angle Spectroscopic Ellipsometry of Nile Red PAGEO5MA Pigment–Polymer Complexes: Dye Distribution

To determine whether Nile Red is uniformly distributed throughout the PAGEO5MA polymer brush film, spectroscopic ellipsometry (SE) data were analysed for a series of samples with varying degrees of dye functionalisation, measured both in the dry state and after equilibration in pH 9 solution. In all cases, the data were modelled using both uniform and graded optical profiles.

Fit quality was assessed using the mean squared error (MSE), which quantifies the deviation between measured and modelled ellipsometric data. During fitting, model parameters are optimised to minimise MSE, however, reductions must be sufficiently large (typically ~25%, threshold commonly adopted in ellipsometric modelling (e.g., J. A. Woollam)) to justify increased model complexity and avoid overfitting. Figure 1 shows (a) the percentage MSE improvement obtained using a graded model relative to a uniform model, and (b) the fitted gradient values and associated uncertainties.

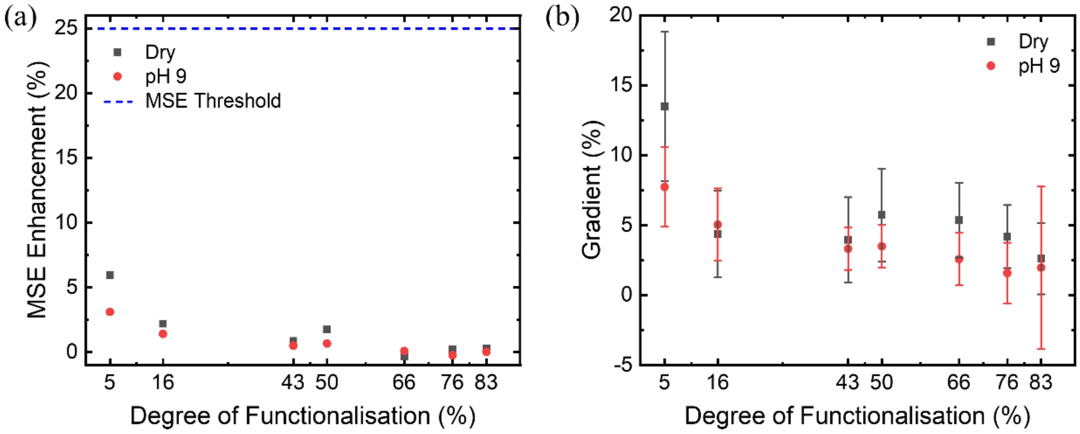

Figure S5 (a) Percentage MSE improvement for graded versus uniform optical models fitted to SE data of Nile Red–functionalised PAGEO5MA polymer brush films, (b) corresponding fitted gradient values with associated uncertainties. Minimal MSE reduction and poorly constrained gradients indicate that inclusion of a gradient does not provide a meaningful improvement to the model.

As shown in Figure 1(a), introduction of a compositional gradient does not provide a meaningful improvement in fit quality. The reduction in MSE is consistently far below the ~25% threshold, with most samples exhibiting <1% improvement and a maximum of ~6% observed for low dye loadings. In these low-loading cases, the minimal optical contrast introduced by the dye limits sensitivity of the SE measurement, and thus small apparent improvements do not reflect physically meaningful gradients.

Furthermore, the fitted gradient parameters (Figure 1(b)) are small in magnitude and poorly constrained, with large associated uncertainties. This indicates that any apparent gradient

is not resolved by the data and lacks physical significance. Under these conditions, inclusion of a gradient constitutes overfitting rather than an accurate physical description.

Accordingly, the uniform layer model provides the most robust and statistically justified representation of the SE data across all samples and conditions investigated. These results provide strong evidence that the dye is distributed throughout the full thickness of the polymer brush film, rather than being confined to a gradient or interfacial region.

Nile Red concentration within PAGEO5MA brushes as a function of degree of functionalization

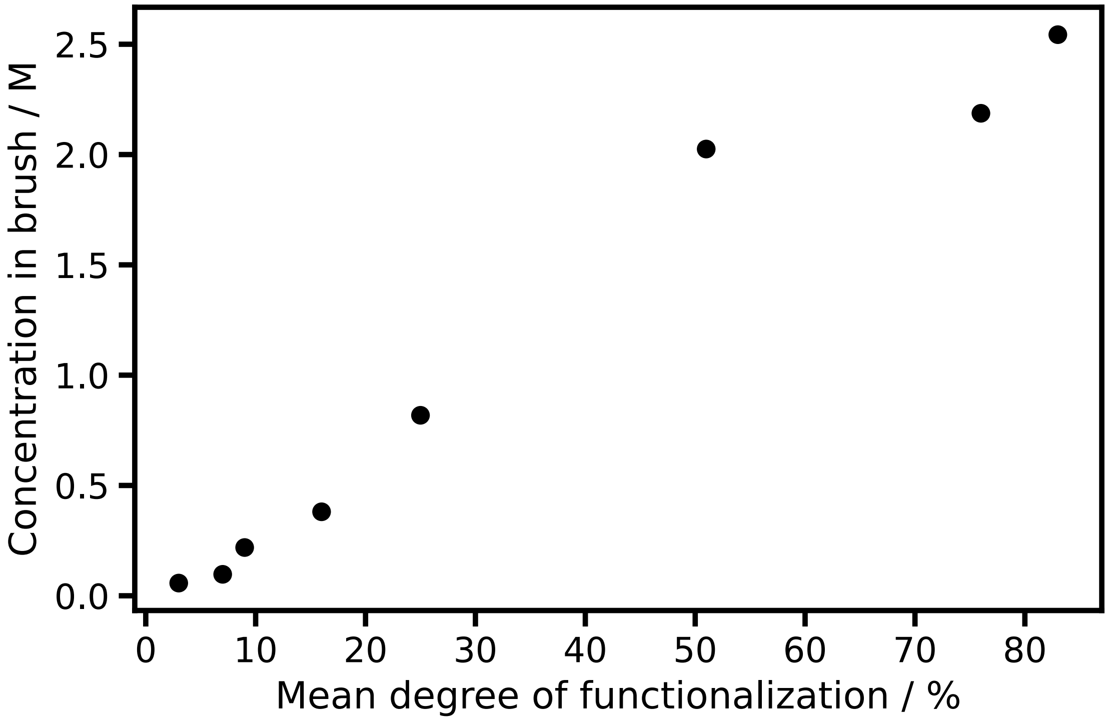

Figure S6. NRet concentration within PAGEO5MA brushes grown from glass coverslips as a function of mean degree of brush functionalization. Dye concentration was calculated from absorption spectra using the Beer-Lambert law. The mean degree of brush functionalization was determined via XPS.

Absorption spectra recorded for Nile Red functionalized PAGEO5MA brushes

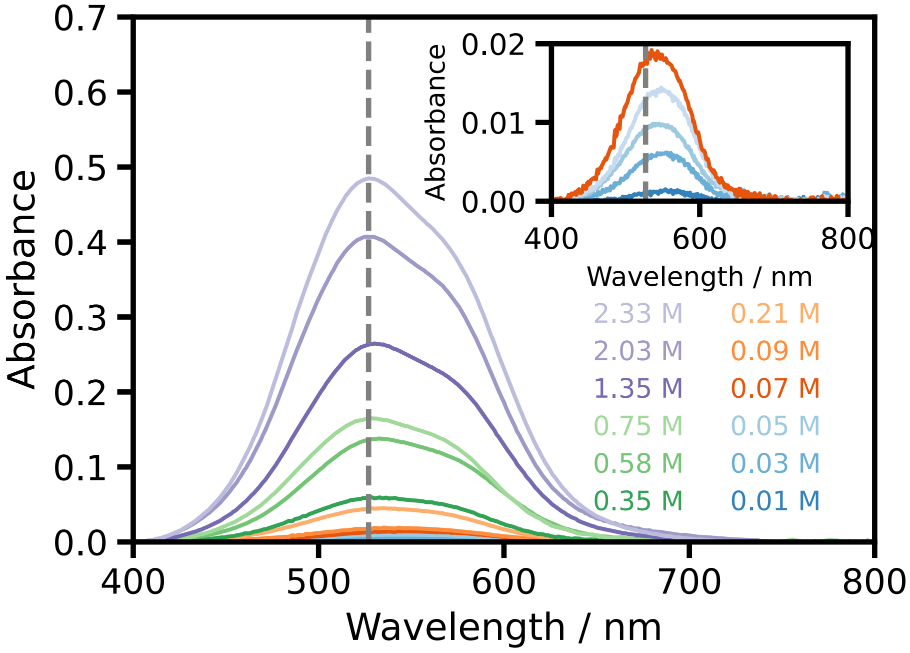

Figure S7. Absorption spectra recorded for NRet-functionalized PAGEO5MA brushes with varying degrees of dye functionalization. Legend indicates the calculated concentration of dye within each brush.

Fluorescence spectra recorded for Nile Red functionalized PAGEO5MA brushes

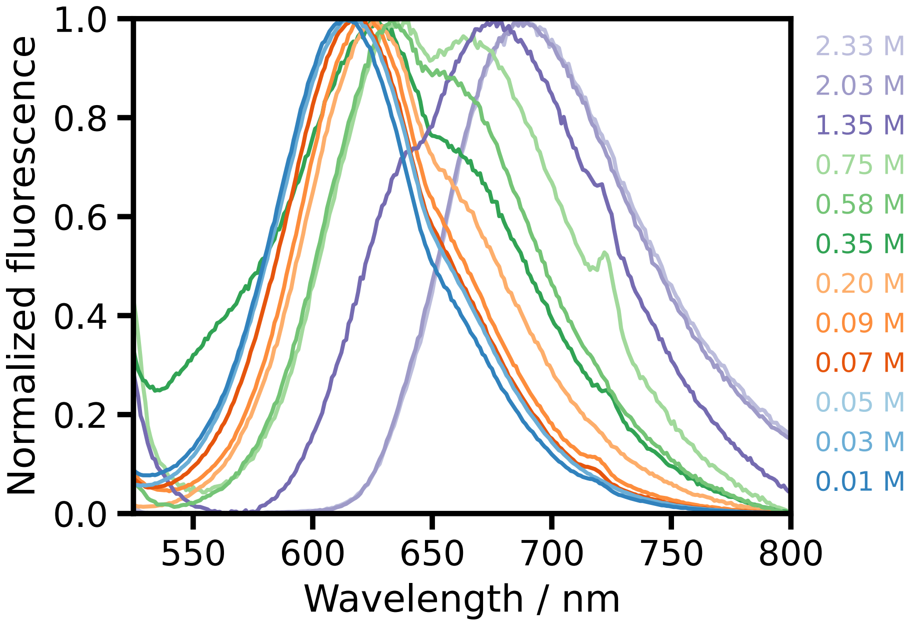

Figure S8. Normalized fluorescence spectra recorded for NRet-functionalized PAGEO5MA brushes with varying degrees of brush functionalization. Legend indicates the calculated concentration of dye within each brush.

References

1 M. Hornum, M. W. Mulberg, M. Szomek, P. Reinholdt, J. R. Brewer, D. Wüstner, J. Kongsted and P. Nielsen, Substituted 9-Diethylaminobenzo[a]phenoxazin-5-ones (Nile Red Analogues): Synthesis and Photophysical Properties, *J Org Chem*, 2021, **86**, 1471–1488.

2 M. S. J. Briggs, I. Bruce, J. N. Miller, C. J. Moody, A. C. Simmonds and E. Swann, Synthesis of functionalised fluorescent dyes and their coupling to amines and amino acids, *J Chem Soc Perkin 1*, 1997, 1051–1058.

3 D. W. Brousmiche, J. M. Serin, J. M. J. Fréchet, G. S. He, T.-C. Lin, S.-J. Chung, P. N. Prasad, R. Kannan and L.-S. Tan, Fluorescence Resonance Energy Transfer in Novel Multiphoton Absorbing Dendritic Structures, *J Phys Chem B*, 2004, **108**, 8592–8600.

4 G. Ghini, L. Lascialfari, C. Vinattieri, S. Cicchi, A. Brandi, D. Berti, F. Betti, P. Baglioni and M. Mannini, Towards a general organogelator: combining a versatile scaffold and an efficient linking process, *Soft Matter*, 2009, **5**, 1863.

5 I. Tosi, M. Segado Centellas, E. Campioli, A. Iagatti, A. Lapini, C. Sissa, L. Baldini, C. Cappelli, M. Di Donato, F. Sansone, F. Santoro and F. Terenziani, Excitation Dynamics in Hetero-bichromophoric Calixarene Systems, *ChemPhysChem*, 2016, **17**, 1686–1706.

6 E. C. Johnson, J. D. Willott, W. M. De Vos, E. J. Wanless and G. B. Webber, Interplay of Composition, pH, and Temperature on the Conformation of Multi-stimulus-responsive Copolymer Brushes: Comparison of Experiment and Theory, *Langmuir*, 2020, **36**, 5765–5777.

7 E. E. Brotherton, E. C. Johnson, M. J. Smallridge, D. B. Hammond, G. J. Leggett and S. P. Armes, Hydrophilic Aldehyde-Functional Polymer Brushes: Synthesis, Characterization, and Potential Bioapplications, *Macromolecules*, 2023, **56**, 2070–2080.

8 T. A. Manz and N. G. Limas, Introducing DDEC6 atomic population analysis: part 1. Charge partitioning theory and methodology, *RSC Adv*, 2016, **6**, 47771–47801.

9 F. Weigend and R. Ahlrichs, Balanced basis sets of split valence, triple zeta valence and quadruple zeta valence quality for H to Rn: Design and assessment of accuracy, *Physical Chemistry Chemical Physics*, 2005, **7**, 3297.

10 Y. Zhao and D. G. Truhlar, The M06 suite of density functionals for main group thermochemistry, thermochemical kinetics, noncovalent interactions, excited states, and transition elements: two new functionals and systematic testing of four M06-class functionals and 12 other functionals, *Theor Chem Acc*, 2008, **120**, 215–241.
